# Transcranial Direct Current Stimulation in neurogenetic syndromes: new treatment perspectives for Down syndrome?

**DOI:** 10.3389/fncel.2024.1328963

**Published:** 2024-02-22

**Authors:** Alessio Faralli, Elisa Fucà, Giulia Lazzaro, Deny Menghini, Stefano Vicari, Floriana Costanzo

**Affiliations:** ^1^Child and Adolescent Neuropsychiatry Unit, Bambino Gesù Children's Hospital (IRCCS), Rome, Italy; ^2^Life Sciences and Public Health Department, Catholic University of Sacred Heart, Rome, Italy

**Keywords:** non-invasive brain stimulation, intellectual disability, excitatory/inhibitory balance, glutamate, GABA, neuroplasticity, trisomy 21

## Abstract

This perspective review aims to explore the potential neurobiological mechanisms involved in the application of transcranial Direct Current Stimulation (tDCS) for Down syndrome (DS), the leading cause of genetically-based intellectual disability. The neural mechanisms underlying tDCS interventions in genetic disorders, typically characterized by cognitive deficits, are grounded in the concept of brain plasticity. We initially present the neurobiological and functional effects elicited by tDCS applications in enhancing neuroplasticity and in regulating the excitatory/inhibitory balance, both associated with cognitive improvement in the general population. The review begins with evidence on tDCS applications in five neurogenetic disorders, including Rett, Prader-Willi, Phelan-McDermid, and Neurofibromatosis 1 syndromes, as well as DS. Available evidence supports tDCS as a potential intervention tool and underscores the importance of advancing neurobiological research into the mechanisms of tDCS action in these conditions. We then discuss the potential of tDCS as a promising non-invasive strategy to mitigate deficits in plasticity and promote fine-tuning of the excitatory/inhibitory balance in DS, exploring implications for cognitive treatment perspectives in this population.

## 1 Introduction

One of the major goals of developmental neuroscience is to broaden the range of strategies aimed at enhancing neural plasticity in neurodevelopmental disorders. Recent findings suggest that brain abnormalities and impaired adaptive and functional neuroplasticity play a role in the pathogenesis of neurodevelopmental disorders with known genetic etiology, such as Down syndrome (DS) (Bartesaghi et al., 2022). Advancements in the knowledge of the fundamental cellular mechanisms of neuroplasticity represent the first step toward the development of innovative potential interventions to improve cognitive and behavioral symptoms.

Emerging evidence points to a dynamic interplay between intrinsic neuronal properties and growth-regulatory cues, which are influenced by external stimuli (Carulli et al., 2011; Chapman et al., 2022). The modulation of the balance between cell-intrinsic mechanisms and extrinsic regulatory molecules creates a *permissive* condition for neuroplasticity. This means that growth-regulatory cues facilitate neuritic remodeling. Conversely, an *instructive* condition for neuroplasticity implies that a cue determines specific features of the phenotype of interest, providing the neurons involved with the right information to promote neuronal connections and support adaptive functions (Carulli et al., 2011; Foscarin et al., 2011).

In summary, *permissive* factors allow *instructive* cues to guide the system toward a specific outcome among the possible ones. The combination of *instructive* and *permissive* factors represents the most promising approach for fostering adaptive neuroplasticity (Woodward, 2010; Branchi and Giuliani, 2021). Moreover, the coordinated interplay of different neurotransmitters and their receptors balances neuronal excitability, promoting network stability during development. The synchronized activity between Gamma-Aminobutyric Acid (GABA) ergic and glutamatergic synapses is essential for preserving an optimal balance in neuronal activity (Chiu et al., 2019). Such interactions are dynamically modulated by activity during development, contributing to Excitation/Inhibition (E/I) balance and to the maturation of neural circuits (Maffei et al., 2017).

A substantial body of scientific research suggests that Non-invasive Brain Stimulation (NiBS) techniques play a role in modulating molecular mechanisms governing neuritic growth for neuronal plasticity and behavioral adaptation (Pelletier et al., 2014; Cirillo et al., 2017). On the other hand, NiBS techniques provide the means to directly and focally stimulate specific brain regions, particularly concerning the balance between inhibitory and excitatory neurotransmitters, although the primary mechanisms of action of different NiBS techniques are distinct (Málly, 2013). Theoretically, NiBS may have the potential to establish a *permissive* environment with implications for the neuroplasticity process.

In this perspective review, we have explored the underlying molecular and biochemical mechanisms of activity-induced plasticity and changes in neuronal transmission associated with a NiBS technique, transcranial Direct Current Stimulation (tDCS), considering its potential application in DS, a genetic disorder of cognition characterized by alterations in plasticity and neural transmission (Bartesaghi et al., 2022).

Given that the atypically developing brain has anatomical and functional differences compared to mature and typically developing brain, it is reasonable to consider that it may respond differently to external stimuli, such as session of stimulation (Garg et al., 2022). Combining pre-clinical research and experimental studies involving participants with DS represents the most effective approach for investigating the potential of tDCS as a treatment tool for cognitive improvement in this population.

### 1.1 Introduction to tDCS technique

NiBS techniques are based on electromagnetic principles and safely induce electrical fields in the brain. In particular, tDCS has been extensively studied and used as a neuromodulation tool, with documented clinical improvements in various neurodevelopmental disorders (Sousa et al., 2022). Research on tDCS efficacy has been conducted in humans (Nitsche et al., 2003), non-human primates (Zanos et al., 2011), rodents *in vivo* (Rohan et al., 2015), and *in vitro* (Fritsch et al., 2010).

tDCS can modulate brain excitability in a polarity-specific manner using a monophasic low-amplitude direct current (0.5 mA−2 mA) (Lefaucheur et al., 2017). The direction of current flow relative to axonal orientation determines whether tDCS has an excitatory (anodal) or inhibitory (cathodal) effect on cortical neurons, by either depolarizing or hyperpolarizing them (Nitsche and Paulus, 2000).

tDCS effects could then be summarized as follows:

*a) Membrane polarization*. tDCS can modify neuronal membrane polarity, altering the probability of generating action potentials by voltage-gated pre and postsynaptic Na+ and Ca^2+^ channels. This process triggers an increase of presynaptic release of excitatory neurotransmitters and postsynaptic calcium influx (Nitsche and Paulus, 2001; Liebetanz et al., 2002; Stagg and Nitsche, 2011), rendering synapses susceptible to long-term depression (LTD) with moderate but prolonged intracellular Ca^2+^ increase and long-term potentiation (LTP) for a short but large Ca^2+^ increase (Lisman, 2001).

*b) Neural transmission*. tDCS has effects on neural transmission, with available evidence showing that anodal tDCS application reduces GABA concentration in the stimulated cerebral cortex, while cathodal tDCS induces impairment in glutamatergic neuronal activity (Stagg et al., 2009; Kim et al., 2014; Zhao et al., 2020). Moreover, tDCS (anodal or cathodal) modulates serotonergic neural activity of the dorsal raphe nucleus by inducing a significant acute inhibition of 5-HT neurons (Cambiaghi et al., 2020). Of note, polarity specific-effects on neurotransmitter concentration need deeper investigation.

*c) Synaptic plasticity*. The effects of tDCS on synaptic plasticity occur at different levels, including calcium dynamics, neurotransmitter release, proteins like receptors, transporters, ion channels and gene expression (Cooke and Bliss, 2006). This suggests that tDCS might exert an effect on the levels of brain-derived neurotrophic factor (BDNF), a modulator of neuronal survival and facilitator of synaptic plasticity (Gray et al., 2013). In particular, tDCS might induce an increase in BDNF concentration when combined with presynaptic stimulation (Fritsch et al., 2010), inducing LTP via BDNF/tyrosine kinase receptor B (TrkB) signaling (Yu et al., 2019). Indeed, Ca2+ signaling and N-metil-D-aspartate (NMDA) glutamatergic receptor activity are the most important phenomena mediating LTP/LTD (Cooke and Bliss, 2006).

## 2 Potential effects of tDCS on plasticity and neuronal transmission

### 2.1 The role of tDCS to boost neuroplasticity

tDCS stimulation appears to be useful in promoting beneficial remodeling of synapses, involving both presynaptic terminals and dendritic spines as postsynaptic elements (Reinhart et al., 2017). Studies in rodents have indicated that tDCS-induced plasticity (Fritsch et al., 2010) is capable of inducing LTP and LTD mediated by NMDA receptors, BDNF and its TrkB, namely BDNF/TrKB pathway (Lu, 2003).

In particular, a critical role for LTP with tDCS (Podda et al., 2016) has been demonstrated in the neurotrophin BDNF. Anodal tDCS over the left hippocampal formation of adult male C57bl/6 mice led to an increase in hippocampal LTP, learning and memory, associated with enhanced acetylation of BDNF promoter, expression of BDNF exons I and IX, and BDNF protein levels. Moreover, enhanced cAMP-response-element-binding protein (CREB) phosphorylation, an important transcription factor for the activation of a number of immediateearly expressing plasticity-related genes, has been observed (Podda et al., 2016). These results suggest that anodal tDCS increases hippocampal LTP with the involvement of BDNF, supporting the therapeutic potential of tDCS for brain diseases associated with impaired neuroplasticity.

On the other hand, human studies exploring tDCS-induced effects on BDNF levels provided contrasting results, with some reports of unmodified peripheral BDNF levels after tDCS intervention (Marangolo et al., 2014; Brunoni et al., 2015) and other reports of alterations in BDNF levels in different neurological and psychiatric conditions, such as Parkinson's disease and schizophrenia (Hadoush et al., 2018; Adam et al., 2021). The inconsistency of results across studies could be explained by methodological differences in detecting BDNF levels in humans as well as by the differences in the selected clinical populations.

Intriguingly, it has been proposed that the changes in cognitive functions induced by tDCS may result from specific interactions between genetically determined network properties and the particular form of stimulation applied (Wiegand et al., 2016). In particular, research on tDCS focused on a single nucleotide polymorphism in the gene encoding BDNF (Val66Met), which affects BDNF expression and secretion (Mallei et al., 2015). Some studies suggest that genetic factors such as Val66Met polymorphism may contribute to the inter-individual variance of tDCS outcomes (Fritsch et al., 2010; Strube et al., 2014; Puri et al., 2015; van der Vliet et al., 2018).

### 2.2 The role of tDCS to modulate excitation/inhibition balance

Animal and human studies consistently demostrate that tDCS can interact with the intrinsic ability of the brain to “restore” balance of neural activity, facilitating the restoration of E/I imbalance and inducing neuroplasticity (Fritsch et al., 2010; Huang et al., 2016, 2017; Hogan et al., 2020). In a study based on adult male C57bl/6 mice, the immediate and after effects of anodal and cathodal tDCS on the primary somatosensory cortex were investigated (Sánchez-León et al., 2021). A polarity-specific bidirectional change of the sensory-evoked potentials, associated with gamma oscillations, was observed. Immunohistochemical analysis corroborated these results, showing changes in Glutamate Decarboxylase (GAD)65/GAD67 immunoreactivity but not in vesicular Glutamate Transporter 1 (vGLUT1) in response to cathodal tDCS (Sánchez-León et al., 2021). In another study, Zhao et al. (2020) employed high definition-anodal/cathodal tDCS over Area 21a of adult male cats' visual cortex. The authors delineated tDCS-induced alterations in neuronal activities, focusing on the concentration and synthesis of GABA and glutamate. After anodal tDCS, the concentration of GABA, but not glutamate, significantly decreased compared to sham group, whereas after cathodal tDCS, the concentration of glutamate, but not GABA, significantly decreased compared to sham group. Moreover, the authors provided evidence of decreased expression of GABA-synthesizing enzymes GAD65 and GAD67 in A21a, whereas no changes were observed for glutamate-synthesizing enzyme glutaminase following anodal tDCS. By contrast, a decrease in both mRNA and protein concentrations of glutaminase in A21a, but not for those of GAD65/GAD67, followed by cathodal tDCS.

In human studies using magnetic resonance spectroscopy, it was found that tDCS alters the levels of multiple neuro-metabolites, particularly glutamate and GABA, specifically but not limited to the site of stimulation (see Chhabra et al., 2021 for a review). In both young and older healthy adults and in patients with neurological and psychiatry disorders, main results confirmed that anodal tDCS induces a reduction in GABA levels (Stagg et al., 2009; Kim et al., 2014; Bachtiar et al., 2015; Antonenko et al., 2017), while cathodal tDCS led to a reduction of glutamate levels (Stagg et al., 2009). However, the baseline level of the neuro-metabolites may predict the outcome after tDCS, as well as the number of stimulation sessions (Chhabra et al., 2021).

## 3 From basic mechanisms toward potential tDCS applications in neurogenetic disorders

Significant alterations in neuroplasticity and E/I balance are believed to underlie various clinical manifestations of neurogenetic disorders. These conditions result from genetic mutations or deletions that disrupt the regulation of brain development (Kolb and Gibb, 2011), often leading to abnormal intellectual, cognitive, and behavioral functioning (Dierssen et al., 2003; Menghini et al., 2011; Anagnostopoulou et al., 2021; Vacca et al., 2023). Emerging treatment strategies for neurogenetic conditions are exploring pharmacological therapies targeting neuroplasticity and neuromodulator balance to enhance cognition and behavior, but their clinical efficacy needs further establishment (Lorenzon et al., 2023). Additionally, NiBS is being considered as a potential treatment for cognitive and behavioral outcome in individuals with neurogenetic disorders, either alone or in combination with other therapies.

### 3.1 tDCS applications in neurogenetic disorders

To explore the therapeutic potential of NiBS in neurogenetic disorders, studies published until December 2023 were reviewed to shed light on the effectiveness of tDCS in improving clinical outcome in five disorders, where genetic factors play a significant role: Rett syndrome (RTT), Prader-Willi syndrome (PWS), Neurofibromatosis type 1 syndrome (NF1), Phelan-McDermid syndrome (PMS), and DS. A summary of the tDCS protocols employed, study design, outcomes, and results of the analyzed studies is reported in Table 1. A detailed description of the main findings in RTT, PWS, NF1 and PMS can be found in the Supplementary material while the description of the main findings in DS can be found in the following paragraph.

**Table 1:** Summary of tDCS studies in individuals with relevant neurogenetic syndromes.

**First author, year**	**Disorder**	**Population characteristics**	**tDCS protocol**						**Study design**	**Outcome and results**
			**Current**	**Duration**	**Session(s)**	**Brain target**	**Montage and conditions**	**Concomitant treatment**		
Fabio et al. (2018)	RTT	a.r.: 29–31 y; *n =* 3; F; IQ = n.a.	2 mA	20 min	10 consecutive days	Broca's area	Anode over the left hemisphere/ cathode over the contralateral right region	Speech rehabilitation	Single subject experimental design ABAA (pre-test, post-test and follow-up)	*Language abilities:* **↑** in the number of vowel/consonant sounds **↑** in the number of words (production and comprehension) *Motor coordination:* **↑** in functional movements *Neurophysiological parameters:* **↑** in the frequency and power of alpha, beta and theta bands
Fabio et al. (2020)	RTT	a.r. 13 - 35 y; *n =* 35; F; IQ = n.a.	2 mA	20 min	10 over a 1-week period	Primary motor cortex	Anode over C3/cathode over the right supraorbital region	Cognitive Empowerment: cognitive-behavioral strategies as imitation procedures, prompting, and generalization	Two groups: non-sham tDCS group and sham tDCS group (pre-test, post-test, follow-up)	*Attention:* **↑** in the attention time *Language abilities:* **↑** in the number of vowel/phonemes sound *Neurophysiological parameters:* **↑** in increase in beta and alpha bands
Azevedo et al. (2017)	PWS	24 y; *n* = 1; M; IQ = n.a./severe ID	2 mA	20 min	10 over a 2-weeks period	Left prefrontal cortex	Anode over the left DLPFC/ cathode over the contralateral right region	None	Single-case study, at-home tDCS protocol: pre-test, post-test, follow-up	**↓** food craving symptoms **↓** behavioral symptoms
Azevedo et al. (2021)	PWS	a.r. 11–35 y; *n =* 12; 4F/8M; IQ = 73.8 ± 10.21	1 mA (a.r. 11–12 y) 2 mA (≥13y)	20 min	10 over a 2-weeks period	Left prefrontal cortex	Anode over the left DLPFC/cathode over the contralateral right region	None	Open label study: tDCS group (pre-test, post-test, follow-up)	**↓** of hyperphagic symptoms **↓** food craving symptoms **↓** behavioral symptoms
Poje et al. (2021)	PWS	a.r. 19–44 y; *n =* 10; 4F/6M; IQ = n-a.	2 mA	30 min	1	DLPFC	F4 for anode/ left supraorbital area for cathode	None	Pre-post design (active/sham)	**↓** in N2 amplitude in the NoGo condition, marginally significant faster reaction times in the Go condition, no effects in accuracy
Bravo et al. (2016)	PWS	a.r. 18–64 y; *n =* 10; 5F/5M; IQ = 62.3 ± 16	2 mA	30 min	5 over 5 consecutive days	DLPFC	F4 for anode/ left supraorbital area	None	Double blind, sham-controlled	*disinhibition, severity and food* *craving*: **↓** symptoms *neuropsychological functioning:* no impact *processing speed:* no impact
Garg et al. (2022)	NF1	a.r. 11–18 y; *n =* 31, 16F/15M; IQ = n.a.	1 mA	15 min	2 over 2 separate days	DLPFC	Anode over F3/ cathode over Cz	None	active/sham two parallel-arm, single blinded, sham-controlled cross-over	**↑** brain activation in the left DLPFC No improvement on working memory
Lopes et al. (2017)	DS	a.r. 6–12 y; *n =* n.a; F/M n.a.; IQ = n.a.	1 mA	20 min	10 sessions, 3 times per week on non-consecutive days	Primary motor cortex	Anodes over C3 and C4/ cathode over the right deltoid muscle	Interactive computer game training involving an upper limb motor training through non-immersive VR motor task	Protocol study for a randomized, sham-controlled, double-blind, clinical trial	EXPECTED: **↑** in upper limb motor functioning **↑** in EEG parameters **↑** in EMG paramiters
Lopes et al. (2020)	DS	8 y; *n* = 1; M; IQ = 70	1 mA	20 min	10 sessions, 3 times a week on non-consecutive days	Primary motor cortex	Anodes over C3 and C4/ cathode over the right deltoid muscle	Interactive computer game training involving an upper limb motor training through non-immersive VR motor task	Case Report: pre-post evaluation and follow-up	*Kinematic variables:* **↑** in movement velocity
Lopes J. B. P. et al. (2022)	DS	Cognitive a.r. 6–12 y; *n =* n.a.; F/M n.a.	1 mA	20 min	10 sessions, 3 times a week on non-consecutive days	DLPFC	Anode over F3 and cathode over the right deltoid muscle	VR game and a manual motor task training	Protocol study for a randomized sham-controlled trial	EXPECTED: enhancement of physical and sensory therapy effects, modulation of muscle activity (EMG) and brain activity (EEG)
Lopes J. et al. (2022)	DS	a.r. 14 - 22 y; *n =* 12; IQ = n.a.; F/M n.a (mean mental age 12 y)	1 mA	20 min	10 sessions, 3 times a week on non-consecutive days	Primary motor cortex	Anodes over C3 and C4/ cathode over the right deltoid muscle	VR computer game training	Observational study (pre-post)	Reorganization of alpha and beta waves
Brunelin et al. (2022)	DS	27 y and 22 y; *n* = 2; 1F/1M	2 mA	20 min	10 over a 2-weeks period	DLPFC	Anode over the left / cathode over the contralateral right region	Both cases under pharmacological treatment	Case report	Both cases recovered from depressive and catatonic symptoms and showed improvement in cognitive functioning
Moyal et al. (2022)	PMS	a.r. 15 – 33 y; *n =* 4; 2F/2M; IQ = n.a.	2 mA	20 min	Case1: 10 Sessions, 2 per day Case2: 14 sessions, 1 per day Case3: 20 sessions, 2 per day Case4: 30 sessions, 2 per day	DLPFC	Anode over the left DLPFC (midway between F3 and FP1) and cathode over the left TPJ (midway between T3 and P3)	Mixed: pharmachological treatment or none	Case Series	In all cases, improvement of spontaneous speech and catatonia symptoms

a.r., age range; M/F, male/females; y, years; n.a., not available; tDCS, transcranial Direct Current Stimulation; mA, milliampere; IQ, Intelligence Quotient; min, minutes; DLPFC, dorsolateral prefrontal cortex; 3D, three dimensions; VR, Virtual Reality; EEG, electroencephalography; EMG, electromyography; DS, Down syndrome; NF1, Neurofibromatosis Type 1; PWS, Prader – Willi syndrome; RTT, Rett syndrome; PMS, Phelan-McDermid syndrome; TPJ, temporo-parietal junction.

The rationale behind the application of tDCS to improve clinical outcome in these neurogenetic disorders varied according to the specific aim and the underlying neurobiology of the disorder. In the case of RTT, the aim was to enhance the excitability of linguistic brain region by applying anodal tDCS over Broca's area, coupled with a cognitive training, to improve language and attention skills (Fabio et al., 2018, 2020). The rationale came from evidence in healthy individuals and in patients with stroke (Fertonani et al., 2010; Cattaneo et al., 2011; Wirth et al., 2011). In the case of PWS, the aim was to enhance prefrontal neural circuit excitability by anodal tDCS with the goal of reducing excessive activity in subcortical structures and, in turn, decreasing hyperphagia (Boggio et al., 2009; Bravo et al., 2016; Azevedo et al., 2017, 2021; Poje et al., 2021). The rationale came from a number of studies in healthy individuals and in patients with substances abuse and food craving (Fregni et al., 2008a,b; Boggio et al., 2009; Goldman et al., 2011). In the case of PMS, tDCS was applied for a modulatory action on the glutamatergic system, particulary on NMDA receptors, to ameliorate catatonia associated to PMS (Moyal et al., 2022). The rationale came from evidence on patients with schizophrenia and bipolar disorder with catatonia (Haroche et al., 2022) and was grounded in the neurobiological hypothesis of PMS, involving haploinsufficiency of SHANK3 associated with NMDA receptor hypofunctionality and catatonia (Kohlenberg et al., 2020). Even in the study on NF1 (Garg et al., 2022), the rationale of tDCS application was grounded in the neurobiological hypothesis of the condition. Anodal tDCS was applied to left dorsolateral prefrontal cortex (DLPFC) to reduce GABA and to improve performance on working memory (WM) tasks, increasing brain activation in the targeted area, since NF1 is characterized by GABAergic over activity and impairment in synaptic plasticity (Costa et al., 2002; Cui et al., 2008; Molosh et al., 2014).

### 3.2 tDCS applications in Down syndrome

DS, the leading cause of genetically-defined intellectual disability (ID), results from the presence of an extra copy of chromosome 21 (de Graaf et al., 2015). The most prominent clinical feature of DS is cognitive impairment, characterized by mild to severe ID, learning deficits, and memory impairment, particularly related to hippocampus-related functions (Pennington et al., 2003; Dierssen et al., 2009; Grieco et al., 2015).

To date, there are very few published data that have applied tDCS in individuals with DS, and most, if not all, have focused on improving motor dysfunction (Lopes et al., 2020; Lopes J. et al., 2022) or addressing psychiatric symptoms (Brunelin et al., 2022), rather than cognitive impairment. The rationale for these interventions often originates from studies involving other clinical populations with motor impairment (Cruz et al., 2005; Nasseri et al., 2015; Santos et al., 2015; Grecco et al., 2017) or psychiatric symptoms and catatonia (Hansbauer et al., 2020; Fregni et al., 2021).

A protocol study was published in 2017 to investigate the effects of anodal tDCS on the primary motor cortex compared to sham stimulation during upper limb motor training involving non-immersive virtual reality (VR) in children with DS (Lopes et al., 2017). Experimental conditions included combined therapy (20 min of 1 mA anodal-tDCS + motor training with VR) and a control group (sham-tDCS with the same electrode montage + motor training with VR). The electrode montage comprised the anode applied over the primary motor cortex and the cathode over the right deltoid muscle, serving as an extra-cephalic reference. The preliminary results of this study were reported as a case report in 2020, demonstrating improvements in various kinematic variables in an eight-year-old child with DS (Lopes et al., 2020). Notably, movement velocity increased during the post-intervention and follow-up evaluations. These findings provide valuable evidence on the combined effects of tDCS on motor functioning in individuals with DS.

The same authors published a clinical trial protocol for sensorimotor improvement involving children with DS to evaluate the application of 20 min of 1 mA tDCS over the DLPFC coupled with sensorimotor training through interactive computer game activities, with the anode placed over F3 and the cathode placed over the right deltoid muscle. This was specifically aimed at evaluating brain activity via electroencephalography (EEG) and muscle activity via electromyography (EMG). The protocol study also included typically developing children as a control group (Trial registration Brazilian Clinical Trials Registry REBEC protocol number RBR-43pk59) (Lopes J. B. P. et al., 2022). Given that individuals with DS are often described as exhibiting clumsiness, particularly in terms of gross movements characterized by slow and less efficient actions (Galli et al., 2010; Rigoldi et al., 2011), researchers have underscored the potential efficacy of tDCS for children with DS. They suggest that the effectiveness of tDCS is further heightened when combined with sensory-motor training.

Additionally, Lopes and colleagues investigated neurophysiological changes in brainwave patterns of children and young adults with DS after 20 min of 1 mA anodal tDCS in the primary motor cortex along with sensorimotor training through interactive computer game activities. In particular, they assessed brain activity in 12 individuals with DS via EEG equipment after tDCS application combined with VR training sessions. Results reported significant differences in event-related desynchronization and event-related synchronization of the alpha and beta rhythms, revealing a reduction in power and frequency and thus a reorganization of the patterns of alpha and beta waves (Lopes J. et al., 2022).

The second area explored for tDCS application in DS is psychiatric symptomatology. Young individuals with DS may experience unexplained cognitive deterioration and unusual regression that may present with loss of skills, mood changes, and repetitive thoughts or behaviors (Santoro et al., 2020). In light of this, tDCS emerges as a promising non-invasive electrical stimulation technique for potential application in psychiatric care for individuals with DS (Brunelin et al., 2022). In both patients described by Brunelin et al. (2022) in their study, sessions of 20 min of 2 mA left anodal/right cathodal tDCS over DLPFC, respectively, enabled patients to be discharged from the hospital, recover from their depressive and catatonic symptoms, and return to their baseline level of functioning within their families. Moreover, cognitive improvement was observed. The treatment was well-received and tolerated, with the only observed side effects after the sessions being temporary itching and redness beneath the electrode placement.

The above-mentioned studies provide support for the neuromodulatory effects and efficacy of tDCS in specific brain regions and networks. Furthermore, these studies suggest that interventions combining physical/cognitive training with tDCS have the potential to induce neuroplasticity and reorganize atypical brain networks, as seen in conditions like DS, enabling them to better adapt to external stimuli demands.

In summary, most studies applying tDCS to neurogenetic disorders have based their approaches on evidence from healthy individuals or other clinical population. However, only a few have specifically hypothesized the application of tDCS based on the neurobiological mechanism where genetic factors play a significant role. While, these studies have provided encouraging results in terms of clinical improvement, there is a crucial need for a deeper understanding of the neurobiological effects of tDCS and its potential for treating neurogenetic disorders.

## 4 Hypothesis of tDCS treatment application in Down syndrome

In this prospective review, we aim to delve into the potential neurobiological mechanisms underlying tDCS application in DS. Typical features of the DS brain that could serve as targets for tDCS treatment include neuroplasticity alterations primarily involving synaptic plasticity, neurogenesis impairment, and a reduced capacity for remodeling.

Imbalances in E/I ratios are linked to abnormalities in the glutamatergic and GABAergic systems (Martínez Cué and Dierssen, 2020; Bartesaghi et al., 2022). Neuroplasticity alterations in DS encompass impaired dendritic maturation, reduced synaptic contacts and dendritic length. A candidate gene implicated in DS synaptic plasticity alterations and cognitive deficits is the dual-specificity tyrosine phosphorylation-regulated kinase 1A (Dyrk1A) gene, associated with ID (Ruiz-Mejias et al., 2016). Its overexpression is thought to lead to defective cortical microarchitecture and E/I imbalance, resulting in altered cognitive abilities (Bull, 2020; Bartesaghi et al., 2022).

Moreover, several evidences indicate dysfunction of GABAergic and glutamatergic systems in the DS brain (Braudeau et al., 2011; Deidda et al., 2015; Kleschevnikov et al., 2017). Previous studies that used Ts65Dn mouse reported a reduction in glutamate levels in the hippocampus and cerebellum, suggesting a general reduction in excitatory neurotransmission (Même et al., 2014; Santin et al., 2014). On the other hand, other studies on murine models of DS focusing on the neurotransmitter GABA showed increased GABAergic terminals density in various brain regions, such as in the dentate gyrus, in the hippocampal formation, particularly the connections between areas CA1-CA3, and in some layers of the cerebral cortex (Martínez-Cu et al., 2013). However, in individuals with DS, GABA acts in an opposite, excitatory manner: instead of reducing the flow, it stimulates it, making it excessive and unregulated due to the imbalance of an electrolyte, the chloride ion (Ben-Ari, 2002; Contestabile et al., 2017). Pharmacological interventions with drugs targeting Cl^−^homeostasis, such as inhibiting Na–K–Cl cotransporter (NKCC1) and/or activating Potassium chloride cotransporter-2 (KCC2), might restore E/I imbalance resulting from an impaired Cl–gradient (Raveendran et al., 2020; Savardi et al., 2023) and rescue cognitive impairment in DS mouse models (Contestabile et al., 2017).

Since tDCS exerts its effects on the brain promoting changes in balance of excitatory and inhibitory neurotransmitters (Sánchez-León et al., 2021) and promoting BDNF-dependent synaptic plasticity (Fritsch et al., 2010; Podda et al., 2016), it could be effective in promoting better adaptive functioning by addressing E/I imbalance and in dendritic morphology and spines in DS by enhancing BDNF expression (Longo et al., 2022). Preclinical studies in DS report an increase in mouse CA1 dendritic spine density after exposure to enriched environment (EE); despite its beneficial effects, EE is not always sufficient to promote long-lasting changes in DS mouse model (Martínez-Cué et al., 2002; Dierssen et al., 2003; Mahoney et al., 2004). Taken together, the results obtained from previous studies (Lopes et al., 2017, 2020) suggest that combined treatment including tDCS and task-specific training could be a potential neuroplasticity enhancing intervention for DS.

In this context, we discuss whether selective effect of depolarization or hyperpolarization induced by tDCS could effectively promote plasticity and modulate imbalance between activation and inhibition signals, reversing electrophysiological and cognitive deficits in people with DS.

Taking advantage of the current knowledge derived from animal and human studies on activity-dependent functional and structural neuroplasticity, it is possible to consider tDCS as a straightforward, safe, and inexpensive technique of NiBS, to induce a powerful effect on cortical modulation. In particular, we propose tDCS as potential treatment applications on promoting compensatory brain reorganization and discussed whether they could work as potential treatment to individuals with DS.

### 4.1 Anodal tDCS

Enhancing excitability through anodal tDCS on brain regions represents the first possibility. The rationale of this approach is grounded in the concept that any kind of brain activity involves modulation of E/I balance at neuronal level. Anodal tDCS, in this context, serves as a brain stimulation method capable of shifting the E/I balance toward excitability.

Grounding from studies in typical population (Harris et al., 2003; Barbati et al., 2022), it is reasonable to anticipate that increasing neuronal excitability in the DS brain could lead to heightened density of glutamatergic terminals. This, in turn, might facilitate the induction of LTP, promote spine head enlargement, foster the formation and stabilization of new spines, and contribute to the mechanisms through which silent synapses can form. Furthermore, anodal tDCS could potentially counterbalance the structural GABAergic effects in DS. Reducing GABA levels through the application of anodal tDCS application may, in effect, diminish the imbalance in the GABA/glutamate ratio and facilitate a reorganization of E/I balance. Additionally, the application of anodal tDCS in individuals with DS might lead to network reorganization, enhanced neurite outgrowth and axonal regeneration, and structural and functional plasticity via BDNF/TrKB pathway (Fehlings and Tator, 1992; Imamura et al., 2006).

Given that tDCS appears to induce a form of plasticity akin to LTP, there is potential for utilizing tDCS to enhance neurocognitive functions in DS by promoting LTP, which seems to be impaired in individuals with DS (Battaglia et al., 2008; Dong et al., 2020). Considering the reduction in NMDA receptors in DS, likely contributing to observed LTP impairment (Bartesaghi, 2022), it is plausible to suggest that anodal tDCS application may help stabilize NMDA receptors and augment Ammino-3-idrossi-5-Metil-4-isossazol-Propionic Acid (AMPA) receptor activation (Henley and Wilkinson, 2013) following calcium influx into the cell. Therefore, anodal tDCS could be administered during specific tasks that induce Hebbian plasticity to harness this phenomenon in DS (Barbati et al., 2022).

### 4.2 Cathodal tDCS

Building on recent knowledge regarding tDCS applications in autism spectrum disorder (Han et al., 2022), cathodal stimulation emerges as another potential protocol in DS. Considering the GABAergic excitatory action observed in DS (Ben-Ari, 2007; Bartesaghi, 2022), cathodal stimulation may prove beneficial in restoring the E/I balance, aiming for a fine-tuned neuronal activity. More specifically, cathodal tDCS could potentially reduce the release of glutamatergic neurotransmitters (Liebetanz et al., 2002; Stagg et al., 2009; Stagg and Nitsche, 2011), thereby influencing plasticity processes with a general decrease of excitatory effect. Improving E/I imbalance in DS brain has the potential to positively impact cognition by moving toward more typical neural signal-to-noise properties. Specifically, if an altered cognitive process is linked to a modified pattern of activity (excessive excitatory signals) in a specific brain region, reducing local excitability could potentially disrupt these crucial patterns. Additionally, this approach may offer a beneficial effect in mitigating the presumed excitotoxic events resulting from heightened glutamate receptor activity in adults with DS (Arai et al., 1996; Cull-Candy et al., 2001; Kaur et al., 2014).

### 4.3 Possible tDCS treatment protocols in DS

A combined approach with tDCS and cognitive and behavioral training appears to be more effective than stimulation alone (Cappelletti et al., 2013; Martin et al., 2014; Mancuso et al., 2016). Strengthening or weakening activation patterns via Hebbian synaptic mechanisms of neuroplasticity, as suggested neural mechanisms of tDCS, can be selectively reinforced by external stimulation, such as cognitive training, speech therapy, motor, attention, and WM training (Jones et al., 2015; Wang et al., 2018; Boroda et al., 2020; Nissim et al., 2020, 2022). For this reason, we suggest potential frameworks and approaches for the practical implementation of tDCS projects in DS that combine tDCS and cognitive and behavioral training, aimed at improving cognition and behavior. Additional tDCS protocols, beyond those already applied in DS for motor deficits and psychiatric aspects, could address language, WM and long-term memory deficits.

#### 4.3.1 Language

Individuals with DS often exhibit a neuropsychological profile characterized by challenges in processing verbal information (Grieco et al., 2015). Limited vocabulary and speech intelligibility represent major issues in DS, and supporting communication is crucial for promoting socialization, improving adaptive abilities, and enhancing overall quality of life (Rodenbusch et al., 2013; Wilkinson and Finestack, 2020). Some evidence suggests the efficacy of interventions for speech and language impairment in younger children with DS (Seager et al., 2022). However, challenges persist, especially for older children and adolescents (Rvachew and Folden, 2018). Incorporating tDCS into traditional interventions for speech and language, which have previously proven successful in DS, could shorten treatment duration and/or amplify outcomes, even in older children and adolescents with DS.

In the context of the language network, several meta-analyses of neuromodulation treatments in language disorders have concluded positive effects (Cotelli et al., 2020; Nissim et al., 2020; Coemans et al., 2021), targeting the left frontal cortex, specifically the left motor cortex and left inferior frontal gyrus (IFG), combined with rehabilitation (Marangolo et al., 2011; Meinzer et al., 2016). Abnormal brain activation in prefrontal cortices and the ventral anterior cingulate (Reynolds Losin et al., 2009; Pujol et al., 2015; Vega et al., 2015), as well as atypical patterns of functional organization for language processing in frontal regions (White et al., 2003; Reynolds Losin et al., 2009; Menghini et al., 2011; Jacola et al., 2014), have been accounted for the reduced linguistic abilities in individuals with DS.

Based on this literature, a potential tDCS treatment protocol for language improvement in DS could target the IFG with the aim of promoting compensatory brain reorganization. Although most of the above-mentioned studies have applied anodal excitatory stimulation, the recent evidence of the effectiveness of inhibitory cathodal stimulation (Han et al., 2022) suggests that cathodal stimulation should also be investigated in linguistic remediation training and multisession tDCS in individuals with DS. The suggested montage could include the active electrode placed on the left IFG (between F5 and F7 of the extended International 10–20 system for EEG electrode placement) cortex and the reference electrode placed above the contralateral shoulder, as previously applied in DS (Lopes et al., 2020; Lopes J. et al., 2022), with a stimulation intensity set at 1–2 mA and a duration of 20 min per session, combined with 20 min−30 min of speech and language training. A minimum of 10 tDCS sessions plus speech and language training sessions could be provided, as suggested in NiBS literature on the remediation of language disorders in genetic syndromes (Fabio et al., 2018, 2020). Promising results for improving communication and language outcomes in DS are offered by interventions employing behavior analytic strategies for language, such as speech, expressive syntax, phonology, and vocabulary training (O'Toole and Chiat, 2006; Neil and Jones, 2018; Smith et al., 2020; Seager et al., 2022), that could be coupled with tDCS sessions.

#### 4.3.2 Short-term memory

Research on DS has extensively examined short-term memory and WM due to their crucial roles in general intelligence and academic achievement (Jarrold and Towse, 2006). Individuals with DS commonly exhibit significant impairments in verbal and visual-spatial span tasks, as well as a general deficit in WM tasks (Lanfranchi et al., 2009, 2010; Costanzo et al., 2013).

One possible tDCS treatment could address memory issues in individuals with DS linked to alterations in the frontal lobe (White et al., 2003; Menghini et al., 2011; Carducci et al., 2013), potentially resulting in a faster and more effective amelioration of memory functions. Neuroimaging studies revealed a widespread effect in cortical activity by anodal tDCS over the DLPFC (Lang et al., 2005) on the entire WM system; furthermore, there is some evidence that WM performance can be improved in a manner dependent on current strength (Teo et al., 2011). Growing evidence suggests that anodal tDCS over the left DLPFC coupled with cathodal right DLPFC, with concurrent memory training, ameliorates cognitive deficits (Aksu et al., 2023), and it could be considered for research scrutinizing the short/long-term efficacy with large samples of individuals with DS.

The stimulation protocol in DS could then include the application of 1–2 mA of anodal tDCS for 20 minutes to DLPFC, with the anodal electrode positioned over F3 according to the 10–20 international system for EEG electrode placement, and the cathode electrode placed over the contralateral supraorbital area. In DS, memory training could be administered through a computerized procedure, focusing on WM enhancement using an n-back task involving verbal, visual, and spatial stimuli, as successfully used in children and adults with DS (Pulina et al., 2015; Lanfranchi et al., 2017). Recognizing the efficacy of programs involving intense repeated practice, treatment sessions could provide sessions at least three times a week, lasting 20 min each.

#### 4.3.3 Long-term memory

An issue of particular relevance is the episodic memory deficits that are a characteristic of cognitive dysfunction in people with DS (Pennington et al., 2003). Diminished long-term memory ability in DS could be linked to abnormalities and dysfunction in the temporal lobe, particularly in the hippocampus (Pennington et al., 2003). A possible investigation could aim to assess the performance of individuals with DS in different forms of episodic and relational memories, where the hippocampus is known to play a central role (Aggleton and Shaw, 1996). Taken individually, tDCS and training have their share of effectiveness, but if used together, tDCS combined with episodic memory training is likely to promote the magnitude of augmenting training-induced cognitive gains in the DS population.

Considering the reduced attention abilities in DS (Lanfranchi et al., 2010; Costanzo et al., 2013) a possible structure for memory training should be designed to be short but frequent, for example, 10-min sessions five times a week for a minimum of 2 weeks. Tasks could involve a cumulative rehearsal strategy during each memory training session. In particular, individuals with DS could be trained with episodic memory tasks using software presentation for different sets of stimuli (e.g., pseudoword-picture pairs) used in different sessions (Antonenko et al., 2017) with concurrent tDCS application. A newly developed training program involving mismatch novelty has been found successful to shape hippocampal responsiveness to synaptic plasticity (Aidil-Carvalho et al., 2017) and the authors suggested the usefulness of this memory training also for DS. The program, based on the exploration of a known environment containing familiar objects, everyday presented in a new location for 2 weeks, enhanced both LTP and LTD in juvenile rats and could represent, after adaptation, another possible training to combine with tDCS in children with DS.

The tDCS montage for long-term memory enhancing could target both the prefrontal cortex and the posterior temporo-parietal junction (TPJ). Some of the studies targeting TPJ applied anodal electrode (1 mA, 20 min) centrally over the left posterior TPJ and cathodal over the right supraorbital area (Antonenko et al., 2017); however, many others found positive results with anodal stimulation to the right TPJ, in both healthy and clinical populations (Flöel et al., 2008; Meinzer et al., 2016; de Sousa et al., 2020). Very recently, a high definition multichannel arrangement to stimulate the right TPJ was found successful to improve hippocampus-dependent spatial memory consolidation in persons with Alzheimer's disease, mild cognitive impairment, and healthy controls (Philippen et al., 2024), and we suggest the utility and potential of this tDCS montage coupled with long-term memory training also for individuals with DS.

## 5 Concluding remarks and future direction of research

### 5.1 tDCS optimal treatment protocols for cognitive improvement in DS

In light of the studies reviewed here, tDCS may emerge as a novel treatment avenue for enhancing cognitive and behavioral functions in neurogenetic disorders. However, it is crucial to acknowledge that the current body of research in this area is limited, and the underlying mechanisms of action remain largely unexplored. Several factors influence tDCS effects, including stimulation intensity, polarity, and the brain's prior activity state (e.g., Romei et al., 2016; Hartwigsen and Silvanto, 2023). While most studies demonstrating cognitive and behavioral improvement have employed anodal excitatory tDCS, recent evidence suggests the potential of cathodal inhibitory stimulation to rebalance E/I activity in neurodevelopmental disorders (Han et al., 2022). Given the complexity of cerebral alterations in DS involving neuroplasticity and E/I imbalance, predicting the effects of different tDCS polarities is challenging. To advance our understanding and develop effective treatment strategies for individuals with DS, it is essential to conduct comparative studies involving both anodal and cathodal tDCS protocols. Moreover, research efforts should strongly promote the development and validation of treatment protocols based on the combination of tDCS and cognitive training, such as speech therapy, attention and memory training, with the aim of shortening treatment duration and/or amplifying outcomes of available interventions for people with DS. These investigations will help identify the optimal parameters for tDCS treatment, laying the groundwork for randomized placebo-controlled trials. Such trials hold significant promise for the translational potential of tDCS-based interventions in the context of DS.

### 5.2 tDCS optimal treatment period for cognitive improvement in DS

The application of NiBS in populations with atypical development, particularly during critical developmental stages, holds the potential to yield more substantial improvements. The developing brain is characterized by a “critical period” during which it is highly receptive to experiences (Knudsen, 2004; Carulli et al., 2010). Extensive literature has demonstrated the potential benefits of tDCS in enhancing motor learning, cognitive, and behavioral outcomes in pediatric populations (Schneider and Hopp, 2011; Costanzo et al., 2016; Van Steenburgh et al., 2017; Esse Wilson et al., 2018; Leffa et al., 2018; Hadar et al., 2020; Jung et al., 2020; Lazzaro et al., 2021, 2022; Han et al., 2022; Salehinejad et al., 2022; Chen et al., 2023). Recent preclinical studies, such as that by Dumontoy et al. (2023), highlight the age-dependent effects of tDCS, underscoring the importance of considering tDCS as a safe and effective intervention in pediatric healthcare. Developing tailored tDCS treatment protocols for individuals with DS during their early years could hold the key to unlocking improved cognitive and functional outcomes for this population.

### 5.3 Multidimensional approach to treatment in DS

**Figure 1:**
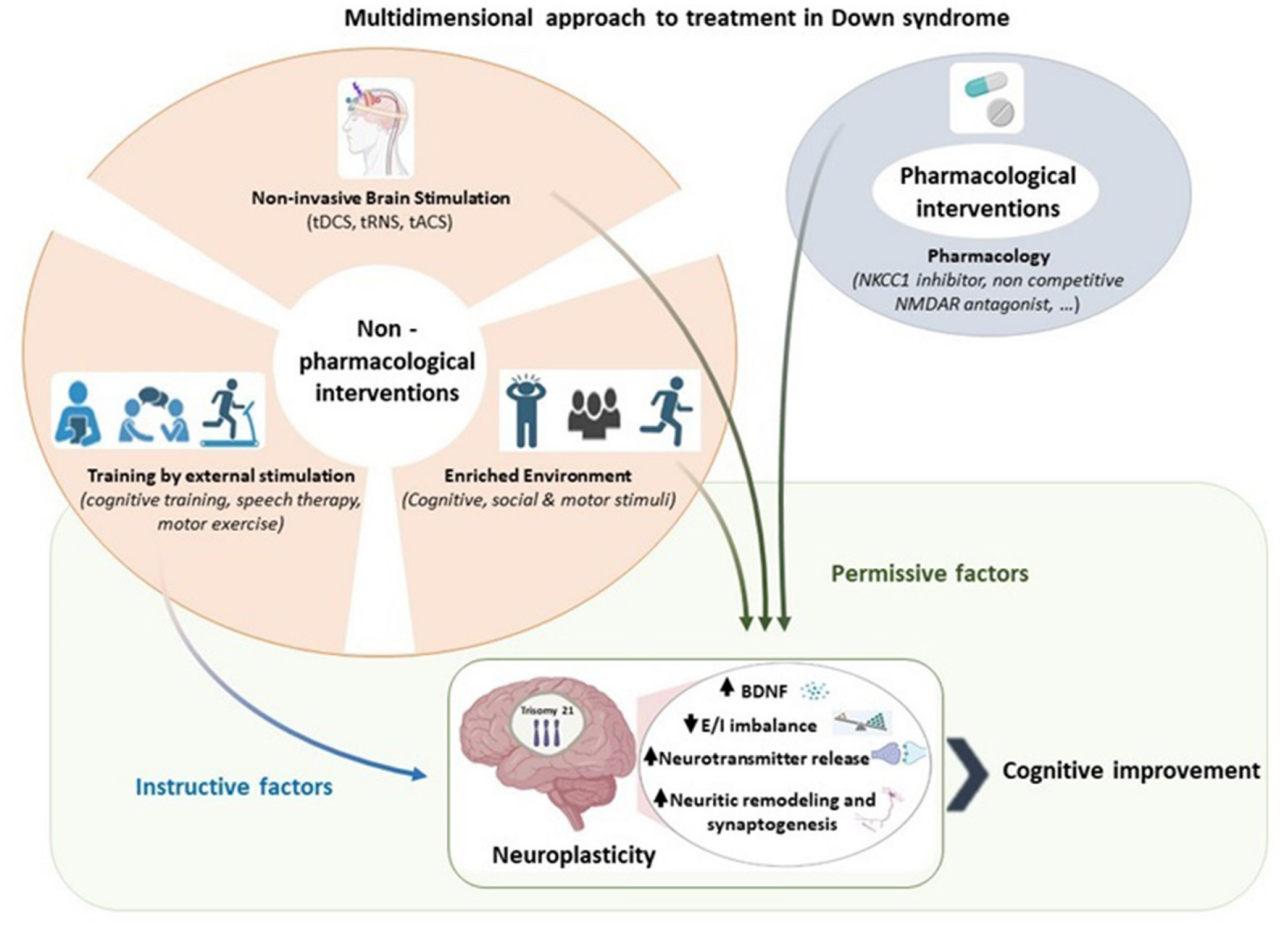
A multidimensional approach to treatment in Down syndrome. Pharmacological and non-pharmacological interventions serve as *permissive* and *instructive* factors of neuroplasticity. We propose that the combination of *instructive* and *permissive* factors offers the most promising approach to promote adaptive neuroplasticity in Down syndrome by enhancing BDNF levels, reducing excitation/inhibition imbalance, increasing neurotransmitter, and facilitating neuritic remodeling and synaptogenesis. Among the *permissive* factors, we highlight the relevance of non-invasive brain stimulation in enabling *instructive* cues to guide Down syndrome brain toward cognitive improvement. Created with BioRender.com.

NiBS techniques combined with a traditional training or rehabilitation paradigm could have higher likelihood of success in stimulating adaptive plasticity in DS. This combined approach as illustrated in Figure 1, hypothesizes a parallel effect by an *instructive* and *permissive* effect, shaping the connectivity in a specific reorganization and dampening plasticity-inhibitory factors, respectively (Faralli et al., 2013; Alia et al., 2021; Zettin et al., 2021). The potential of this approach lies in its ability to improve functioning by addressing altered E/I balance and neuroplasticity (Bavelier et al., 2010). By strengthening or weakening activation patterns through Hebbian synaptic mechanisms of neuroplasticity, tDCS may modulate external stimulation, such as cognitive, speech and motor training, selectively reinforcing their effects (Jones et al., 2015; Wang et al., 2018; Boroda et al., 2020; Nissim et al., 2020, 2022).

Considering the underlying mechanisms triggered by the use of endogenous and exogenous intervention, such as pharmacological, EE and NiBS respectively, our purpose is to use them complementary.

### 5.4 Future direction of research

While this mini-review primarily focuses on tDCS, it is important to note that NiBS encompasses other non-invasive procedures applied both in healthy individuals and in neuropsychiatric disorders. Transcranial Alternating Current Stimulation (tACS) and transcranial Random Noise Stimulation (tRNS) are among these methods, capable of non-invasively modulating brain oscillations (Antal and Herrmann, 2016; Boetzel and Herrmann, 2021). Exploring the application of these recent NiBS techniques to address the electroencephalography abnormalities observed in DS (Babiloni et al., 2010; Velikova et al., 2011; Hamburg et al., 2021) holds promise for more comprehensive therapeutic approaches.

To provide a personalized therapeutic approach, it is imperative to develop a research model that bridges the gap between animal and human studies, facilitating the integration of clinical applications and personalized interventions for cognitive functioning in DS using tDCS. This integrative approach should consider the state-of-the-art neuronal parameters implicated in DS, such as elevated intracellular Cl– levels, disrupted E/I balance, and neuroplasticity abnormalities. Preclinical research would enable us to directly assess changes related to cortical E/I balance and synaptic plasticity after tDCS in DS, which can be validated through immunohistological analyses, including histological markers such as vGLUT1 or Vesicular GABA transporter (VGAT).

In summary, a multidimensional approach combining drugs, cognitive training, environmental stimulation, and NiBS techniques like tDCS could provide effective strategies for promoting brain plasticity, addressing cognitive aspects such as expressive language, memory, and executive functions, and above all enhancing the quality of life of individuals with DS.

## Author contributions

AF: Writing—original draft, Writing—review & editing, Conceptualization. EF: Writing—original draft, Writing—review & editing. GL: Writing—original draft, Writing—review & editing. DM: Writing—original draft, Writing—review & editing. SV: Writing—original draft, Writing—review & editing. FC: Writing—original draft, Writing—review & editing, Conceptualization, Supervision.

## References

[B1] Adam O. Psomiades M. Rey R. Mandairon N. Suaud-Chagny M. F. Mondino M.. (2021). Frontotemporal transcranial direct current stimulation decreases serum mature brain-derived neurotrophic factor in schizophrenia. Brain Sci. 11:662. 10.3390/brainsci11050662 34069556 PMC8160668

[B2] Aggleton J. P. Shaw C. (1996). Amnesia and recognition memory: a re-analysis of psychometric data. Neuropsychologia 34, 51–62. 10.1016/0028-3932(95)00150-6 8852693

[B3] Aidil-Carvalho M. F. Carmo A. J. S. Ribeiro J. A. Cunha-Reis D. (2017). Mismatch novelty exploration training enhances hippocampal synaptic plasticity: a tool for cognitive stimulation? Neurobiol. Learn Mem. 12, 240–250. 10.1016/j.nlm.2017.09.004 28893669

[B4] Aksu S. Hasirci Bayir B. R. Sayman C. Soyata A. Z. Boz G. Karamürsel S. (2023). Working memory improvement after transcranial direct current stimulation paired with working memory training in diabetic peripheral neuropathy. Appl. Neuropsychol. Adult 12, 1–14. 10.1080/23279095.2022.2164717 36630270

[B5] Alia C. Cangi D. Massa V. Salluzzo M. Vignozzi L. Caleo M.. (2021). Cell-to-cell interactions mediating functional recovery after stroke. Cells 10:3050. 10.3390/cells10113050 34831273 PMC8623942

[B6] Anagnostopoulou A. Styliadis C. Kartsidis P. Romanopoulou E. Zilidou V. Karali C.. (2021). Computerized physical and cognitive training improves the functional architecture of the brain in adults with down syndrome: a network science EEG study. Netw. Neurosci. 5, 274–294. 10.1162/netn_a_00177 33688615 PMC7935030

[B7] Antal A. Herrmann C. S. (2016). Transcranial alternating current and random noise stimulation: possible mechanisms. Neural Plast. 2016:3616807. 10.1155/2016/3616807 27242932 PMC4868897

[B8] Antonenko D. Schubert F. Bohm F. Ittermann B. Aydin S. Hayek D.. (2017). tDCS-induced modulation of gaba levels and resting-state functional connectivity in older adults. J Neurosci. 37, 4065–4073. 10.1523/JNEUROSCI.0079-17.2017 28314813 PMC6596583

[B9] Arai Y. Mizuguchi M. Takashima S. (1996). Excessive glutamate receptor 1 immunoreactivity in adult Down syndrome brains. Pediatr. Neurol. 15, 203–206. 10.1016/S0887-8994(96)00167-1 8916156

[B10] Azevedo C. Gomes J. S. Trevizol A. P. Dias Á. M. Cordeiro Q. (2017). At-home transcranial direct current stimulation in prader-willi syndrome with severe intellectual disability: a case study. J ECT. 33, e29–e30. 10.1097/YCT.0000000000000409 28383347

[B11] Azevedo C. C. Trevizol A. P. Gomes J. S. Akiba H. Franco R. R. Simurro P. B.. (2021). Transcranial direct current stimulation for Prader-Willi syndrome. *J ECT*. 37, 58–63. 10.1097/YCT.0000000000000722 33009217

[B12] Babiloni C. Albertini G. Onorati P. Muratori C. Buffo P. Condoluci C.. (2010). Cortical sources of EEG rhythms are abnormal in down syndrome. Clin. Neurophysiol. 121, 1205–1212. 10.1016/j.clinph.2010.02.155 20362500

[B13] Bachtiar V. Near J. Johansen-Berg H. Stagg C. J. (2015). Modulation of GABA and resting state functional connectivity by transcranial direct current stimulation. Elife 4, e08789. 10.7554/eLife.08789 26381352 PMC4654253

[B14] Barbati S. A. Podda M. V. Grassi C. (2022). Tuning brain networks: The emerging role of transcranial direct current stimulation on structural plasticity. Front. Cell Neurosci. 16:945777. 10.3389/fncel.2022.945777 35936497 PMC9351051

[B15] Bartesaghi R. (2022). Brain circuit pathology in Down syndrome: from neurons to neural networks. Rev. Neurosci. 17, 1–14. 10.1515/revneuro-2022-0067 36170842

[B16] Bartesaghi R. Vicari S. Mobley W. C. (2022). Prenatal and postnatal pharmacotherapy in down syndrome: the search to prevent or ameliorate neurodevelopmental and neurodegenerative disorders. Annu. Rev. Pharmacol. Toxicol. 62, 211–233. 10.1146/annurev-pharmtox-041521-103641 34990205 PMC9632639

[B17] Battaglia F. Quartarone A. Rizzo V. Ghilardi M. F. Di Rocco A. Tortorella G.. (2008). Early impairment of synaptic plasticity in patients with Down's syndrome. Neurobiol. Aging. 29, 1272–1275. 10.1016/j.neurobiolaging.2007.02.025 17399853

[B18] Bavelier D. Levi D. M. Li R. W. Dan Y. Hensch T. K. (2010). Removing brakes on adult brain plasticity: from molecular to behavioral interventions. J. Neurosci. 30, 14964–14971. 10.1523/JNEUROSCI.4812-10.2010 21068299 PMC2992973

[B19] Ben-Ari Y. (2002). Excitatory actions of gaba during development: the nature of the nurture. Nat. Rev. Neurosci. 3, 728–739. 10.1038/nrn920 12209121

[B20] Ben-Ari Y. (2007). GABA excites and sculpts immature neurons well before delivery: modulation by GABA of the development of ventricular progenitor cells. Epilepsy Curr. 7, 167–9. 10.1111/j.1535-7511.2007.00214.x 18049728 PMC2096719

[B21] Boetzel C. Herrmann C. S. (2021). Potential targets for the treatment of ADHD using transcranial electrical current stimulation. Prog. Brain Res. 264, 151–170. 10.1016/bs.pbr.2021.01.011 34167654

[B22] Boggio P. S. de Macedo E. C. Schwartzman J. S. Brunoni D. Teixeira M. C. Fregni F.. (2009). Transcranial direct current stimulation: a novel approach to control hyperphagia in Prader-Willi syndrome. J. Child Neurol. 24, 642–643. 10.1177/0883073808322339 19406762

[B23] Boroda E. Sponheim S. R. Fiecas M. Lim K. O. (2020). Transcranial direct current stimulation (tDCS) elicits stimulus-specific enhancement of cortical plasticity. Neuroimage 211:116598. 10.1016/j.neuroimage.2020.116598 32032738

[B24] Branchi I. Giuliani A. (2021). Shaping therapeutic trajectories in mental health: instructive vs. permissive causality. Eur. Neuropsychopharmacol. 43, 1–9. 10.1016/j.euroneuro.2020.12.001 33384216

[B25] Braudeau J. Delatour B. Duchon A. Pereira P. L. Dauphinot L. de Chaumont F.. (2011). Specific targeting of the GABA-A receptor α5 subtype by a selective inverse agonist restores cognitive deficits in Down syndrome mice. J. Psychopharmacol. 25, 1030–1042. 10.1177/0269881111405366 21693554 PMC3160204

[B26] Bravo G. L. Poje A. B. Perissinotti I. Marcondes B. F. Villamar M. F. Manzardo A. M.. (2016). Transcranial direct current stimulation reduces food-craving and measures of hyperphagia behavior in participants with Prader-Willi syndrome. Am. J. Med. Genet. B. Neuropsychiatr. Genet. 171B, 266–275. 10.1002/ajmg.b.32401 26590516 PMC6668339

[B27] Brunelin J. Adam O. Favre E. Prange S. Zante E. Demily C.. (2022). Noninvasive electrical stimulation for psychiatric care in Down syndrome. Brain Stimul. 15, 678–679. 10.1016/j.brs.2022.04.012 35470018

[B28] Brunoni A. R. Baeken C. Machado-Vieira R. Gattaz W. F. Vanderhasselt M. A. (2015). BDNF blood levels after non-invasive brain stimulation interventions in major depressive disorder: a systematic review and meta-analysis. World J. Biol. Psychiatr. 16, 114–122. 10.3109/15622975.2014.958101 25264290

[B29] Bull M. J. (2020). Down syndrome. N. Engl. J. Med. 382, 2344–2352. 10.1056/NEJMra1706537 32521135

[B30] Cambiaghi M. Buffelli M. Masin L. Valtorta F. Comai S. (2020). Transcranial direct current stimulation of the mouse prefrontal cortex modulates serotonergic neural activity of the dorsal raphe nucleus. Brain stimul. 13, 548–550. 10.1016/j.brs.2020.01.012 32289674

[B31] Cappelletti M. Gessaroli E. Hithersay R. Mitolo M. Didino D. Kanai R.. (2013). Transfer of cognitive training across magnitude dimensions achieved with concurrent brain stimulation of the parietal lobe. J. Neurosci. 33, 14899–14907. 10.1523/JNEUROSCI.1692-13.2013 24027289 PMC3771029

[B32] Carducci F. Onorati P. Condoluci C. Di Gennaro G. Quarato P. P. Pierallini A.. (2013). Whole-brain voxel-based morphometry study of children and adolescents with Down syndrome. Funct. Neurol. 28, 19–28. 10.11138/FNeur/2013.28.1.019 23731912 PMC3812718

[B33] Carulli D. Foscarin S. Rossi F. (2011). Activity-dependent plasticity and gene expression modifications in the adult CNS. Front. Mol. Neurosci.. 28:50. 10.3389/fnmol.2011.00050 22144945 PMC3226246

[B34] Carulli D. Pizzorusso T. Kwok J. C. Putignano E. Poli A. Forostyak S.. (2010). Anis lacking link protein have attenuated perineuronal nets and persistent plasticity. Brain 133, 2331–2347. 10.1093/brain/awq145 20566484

[B35] Cattaneo Z. Pisoni A. Papagno C. (2011). Transcranial direct current stimulation over Broca's region improves phonemic and semantic fluency in healthy individuals. Neuroscience 183, 64–70. 10.1016/j.neuroscience.2011.03.058 21477637

[B36] Chapman C. A. Nuwer J. L. Jacob T. C. (2022). The Yin and Yang of GABAergic and glutamatergic synaptic plasticity: opposites in balance by crosstalking mechanisms. Front. Synaptic. Neurosci. 19:911020. 10.3389/fnsyn.2022.911020 35663370 PMC9160301

[B37] Chen H. Y. Yang C. Y. Hsieh T. H. Peng C. W. Chuang L. L. Chang Y. L.. (2023). Effects of transcranial direct current stimulation on improving performance of delayed- reinforcement attentional set-shifting tasks in attention-deficit/hyperactivity disorder rat model. Behav. Brain Res. 2:114145. 10.1016/j.bbr.2022.114145 36206819

[B38] Chhabra H. Thimmashetty V. H. Shivakumar V. Venkatasubramanian G. Narayanswamy J. C. (2021). Effect of transcranial direct current stimulation on in-vivo assessed neuro-metabolites through magnetic resonance spectroscopy: a systematic review. Acta Neuropsychiatr. 33, 242–253. 10.1017/neu.2021.14 33926587

[B39] Chiu C. Q. Barberis A. Higley M. J. (2019). Preserving the balance: diverse forms of long-term GABAergic synaptic plasticity. Nat. Rev. Neurosci. 20, 272–281. 10.1038/s41583-019-0141-5 30837689

[B40] Cirillo G. Di Pino G. Capone F. Ranieri F. Florio L. Todisco V. V.. (2017). Neurobiological after-effects of non-invasive brain stimulation. Brain Stimul. 10, 1–18. 10.1016/j.brs.2016.11.009 27931886

[B41] Coemans S. Struys E. Vandenborre D. Wilssens I. Engelborghs S. Paquier P.. (2021). A Systematic Review of Transcranial Direct Current Stimulation in Primary Progressive Aphasia: methodological considerations. Front. Aging Neurosci. 13:710818. 10.3389/fnagi.2021.710818 34690737 PMC8530184

[B42] Contestabile A. Magara S. Cancedda L. (2017). The GABAergic hypothesis for cognitive disabilities in down syndrome. Front Cell Neurosci. 11:54. 10.3389/fncel.2017.00054 28326014 PMC5339239

[B43] Cooke S. F. Bliss T. V. (2006). Plasticity in the human central nervous system. Brain 129:1659–1673. 10.1093/brain/awl082 16672292

[B44] Costa R. M. Federov N. B. Kogan J. H. Murphy G. G. Stern J. Ohno M.. (2002). Mechanism for the learning deficits in a mouse model of neurofibromatosis type 1. Nature 415, 526–530. 10.1038/nature711 11793011

[B45] Costanzo F. Varuzza C. Menghini D. Addona F. Gianesini T. Vicari S.. (2013). Executive functions in intellectual disabilities: a comparison between Williams syndrome and Down syndrome. Res. Dev. Disab. 34, 1770–1780. 10.1016/j.ridd.2013.01.024 23501586

[B46] Costanzo F. Varuzza C. Rossi S. Sdoia S. Varvara P. Oliveri M.. (2016). Reading changes in children and adolescents with dyslexia after transcranial direct current stimulation. Neuroreport 23, 295–300. 10.1097/WNR.0000000000000536 26848997

[B47] Cotelli M. Manenti R. Ferrari C. Gobbi E. Macis A. Cappa S. F.. (2020). Effectiveness of language training and non-invasive brain stimulation on oral and written naming performance in Primary Progressive Aphasia: a meta-analysis and systematic review. Neurosci. Biobehav. Rev. 108, 498–525. 10.1016/j.neubiorev.2019.12.003 31811834

[B48] Cruz E. G. Waldinger H. C. Kamper D. G. (2005). Kinetic and kinematic workspaces of the index finger following stroke. Brain. 128(Pt 5), 1112-1121. 10.1093/brain/awh432 15743873

[B49] Cui Y. Costa R. M. Murphy G. G. Elgersma Y. Zhu Y. Gutmann D. H.. (2008). Neurofibromin regulation of ERK signaling modulates GABA release and learning. Cell 135, 549–560. 10.1016/j.cell.2008.09.060 18984165 PMC2673196

[B50] Cull-Candy S. Brickley S. Farrant M. (2001). NMDA receptor subunits: diversity, development and disease. Curr. Opin. Neurobiol. 11, 327–335. 10.1016/S0959-4388(00)00215-4 11399431

[B51] de Graaf G. Buckley F. Skotko B. G. (2015). Estimates of the live births, natural losses, and elective terminations with Down syndrome in the United States. Am. J. Med. Genet A. 167A, 756–767. 10.1002/ajmg.a.37001 25822844

[B52] de Sousa A. V. C. Grittner U. Rujescu D. Külzow N. Flöel A. (2020). Impact of 3-day combined anodal transcranial direct current stimulation-visuospatial training on object-location memory in healthy older adults and patients with mild cognitive impairment. J. Alzheimers Dis. 75, 223–244. 10.3233/JAD-191234 32280093 PMC7306891

[B53] Deidda G. Parrini M. Naskar S. Bozarth I. F. Contestabile A. Cancedda L.. (2015). Reversing excitatory GABAAR signaling restores synaptic plasticity and memory in a mouse model of Down syndrome. Nat. Med. 21, 318–326. 10.1038/nm.3827 25774849

[B54] Dierssen M. Benavides-Piccione R. Martínez-Cu,é C. Estivill X. Flórez J. Elston G. N.. (2003). Alterations of neocortical pyramidal cell phenotype in the Ts65Dn mouse model of Down syndrome: effects of environmental enrichment. Cereb. Cortex. 13, 758–764. 10.1093/cercor/13.7.758 12816891

[B55] Dierssen M. Herault Y. Estivill X. (2009). Aneuploidy: from a physiological mechanism of variance to Down syndrome. Physiol. Rev. 89, 887–920. 10.1152/physrev.00032.2007 19584316

[B56] Dong Y. Xiong M. Chen Y. Tao Y. Li X. Bhattacharyya A.. (2020). Plasticity of synaptic transmission in human stem cell-derived neural networks. iScience. 23:100829. 10.1016/j.isci.2020.100829 31981924 PMC6993006

[B57] Dumontoy S. Ramadan B. Risold P. Y. Pedron S. Houdayer C. Etiévant A.. (2023). Repeated anodal transcranial direct current stimulation (RA-tDCS) over the left frontal lobe increases bilateral hippocampal cell proliferation in young adult but not middle-aged female mice. Int. J. Mol. Sci. 24:8750. 10.3390/ijms24108750 37240095 PMC10218697

[B58] Esse Wilson J. Trumbo M. C. Wilson J. K. Tesche C. D. (2018). Transcranial direct current stimulation (tDCS) over right temporoparietal junction (rTPJ) for social cognition and social skills in adults with autism spectrum disorder (ASD). J. Neural. Transm. 125, 1857–1866. 10.1007/s00702-018-1938-5 30341695

[B59] Fabio R. A. Gangemi A. Capri T. Budden S. Falzone A. (2018). Neurophysiological and cognitive effects of transcranial direct current stimulation in three girls with rett syndrome with chronic language impairments. Res. Dev. Disabil. 76, 76–87. 10.1016/j.ridd.2018.03.008 29587149

[B60] Fabio R. A. Gangemi A. Semino M. Vignoli A. Canevini M. P. Priori A.. (2020). Effects of combined transcranial direct current stimulation with cognitive training in girls with rett syndrome. Brain Sci. 10:276. 10.3390/brainsci10050276 32370253 PMC7287589

[B61] Faralli A. Bigoni M. Mauro A. Rossi F. Carulli D. (2013). Noninvasive strategies to promote functional recovery after stroke. Neural. Plast. 2013:854597. 10.1155/2013/854597 23864962 PMC3707231

[B62] Fehlings M. G. Tator C. H. (1992). The effect of direct current field polarity on recovery after acute experimental spinal cord injury. Brain Res. 579, 32–42. 10.1016/0006-8993(92)90738-U 1623405

[B63] Fertonani A. Rosini S. Cotelli M. Rossini P. M. Miniussi C. (2010). Naming facilitation induced by transcranial direct current stimulation. Behav. Brain Res. 208, 311–318. 10.1016/j.bbr.2009.10.030 19883697

[B64] Flöel A. Rösser N. Michka O. Knecht S. Breitenstein C. (2008). Noninvasive brain stimulation improves language learning. J. Cogn. Neurosci. 20, 1415–1422. 10.1162/jocn.2008.20098 18303984

[B65] Foscarin S. Ponchione D. Pajaj E. Leto K. Gawlak M. Wilczynski G. M.. (2011). Experience-dependent plasticity and modulation of growth regulatory molecules at central synapses. PLoS ONE 6:e16666. 10.1371/journal.pone.0016666 21304956 PMC3031615

[B66] Fregni F. El-Hagrassy M. M. Pacheco-Barrios K. Carvalho S. Leite J. Simis M.. (2021). Neuromodulation Center Working Group. Evidence-based guidelines and secondary meta-analysis for the use of transcranial direct current stimulation in neurological and psychiatric disorders. Int. J. Neuropsychopharmacol. 24, 256–313. 10.1093/ijnp/pyaa051 32710772 PMC8059493

[B67] Fregni F. Liguori P. Fecteau S. Nitsche M. A. Pascual-Leone A. Boggio P. S.. (2008b). Cortical stimulation of the prefrontal cortex with transcranial direct current stimulation reduces cue-provoked smoking craving: a randomized, sham-controlled study. J. Clin. Psychiatr. 69, 32–40. 10.4088/JCP.v69n0105 18312035

[B68] Fregni F. Orsati F. Pedrosa W. Fecteau S. Tome F. A. Nitsche M. A.. (2008a). Transcranial direct current stimulation of the prefrontal cortex modulates the desire for specific foods. Appetite 51, 34–41. 10.1016/j.appet.2007.09.016 18243412 PMC3541023

[B69] Fritsch B. Reis J. Martinowich K. Schambra H. M. Ji Y. Cohen L. G.. (2010). Direct current stimulation promotes BDNF-dependent synaptic plasticity: potential implications for motor learning. Neuron 29, 198–204. 10.1016/j.neuron.2010.03.035 20434997 PMC2864780

[B70] Galli M. Rigoldi C. Brunner R. Virji-Babl N. Giorgio A. (2010). Joint stiffness and gait pattern evaluation in children with Down syndrome. Gait Posture. 28, 502–506. 10.1016/j.gaitpost.2008.03.00 18455922

[B71] Garg S. Williams S. Jung J. Pobric G. Nandi T. Lim B.. (2022). Non-invasive brain stimulation modulates GABAergic activity in neurofibromatosis 1. Sci Rep. 12:18297. 10.1038/s41598-022-21907-9 36316421 PMC9622815

[B72] Goldman R. L. Borckardt J. J. Frohman H. A. O'Neil P. M. Madan A. Campbell L. K.. (2011). Prefrontal cortex transcranial direct current stimulation (tDCS) temporarily reduces food cravings and increases the self-reported ability to resist food in adults with frequent food craving. Appetite 56, 741–746. 10.1016/j.appet.2011.02.013 21352881

[B73] Gray J. D. Milner T. A. McEwen B. S. (2013). Dynamic plasticity: the role of glucocorticoids, brain-derived neurotrophic factor and other trophic factors. Neuroscience 239, 214–227. 10.1016/j.neuroscience.2012.08.034 22922121 PMC3743657

[B74] Grecco L. A. Oliveira C. S. Duarte N. A. Lima V. L. Zanon N. Fregni F.. (2017). Cerebellar transcranial direct current stimulation in children with ataxic cerebral palsy: a sham-controlled, crossover, pilot study. Dev. Neurorehabil. 20, 142–148. 10.3109/17518423.2016.1139639 27003795

[B75] Grieco J. Pulsifer M. Seligsohn K. Skotko B. Schwartz A. (2015). Down syndrome: cognitive and behavioral functioning across the lifespan. Am. J. Med. Genet. C. Semin. Med. Genet. 169, 135–149. 10.1002/ajmg.c.31439 25989505

[B76] Hadar R. Winter R. Edemann-Callesen H. Wieske F. Habelt B. Khadka N.. (2020). Prevention of schizophrenia deficits via non-invasive adolescent frontal cortex stimulation in rats. Mol. Psychiatr. 25, 896–905. 10.1038/s41380-019-0356-x 30692610

[B77] Hadoush H. Banihani S. A. Khalil H. Al-Qaisi Y. Al-Sharman A. Al-Jarrah M.. (2018). Dopamine, BDNF and motor function postbilateral anodal transcranial direct current stimulation in Parkinson's disease. Neurodegener. Dis. Manag. 8, 171–179. 10.2217/nmt-2017-0048 29888648

[B78] Hamburg S. Bush D. Strydom A. Startin C. M. (2021). Comparison of resting-state EEG between adults with Down syndrome and typically developing controls. J. Neurodev. Disord. 13, 1–11. 10.1186/s11689-021-09392-z 34649497 PMC8518326

[B79] Han Y. M. Y. Chan M. M. Y. Shea C. K. S. Lai O. L. Krishnamurthy K. Cheung M. C.. (2022). Neurophysiological and behavioral effects of multisession prefrontal tDCS and concurrent cognitive remediation training in patients with autism spectrum disorder (ASD): a double-blind, randomized controlled fNIRS study. Brain Stimul. 15, 414–425. 10.1016/j.brs.2022.02.004 35181532

[B80] Hansbauer M. Wagner E. Strube W. Röh A. Padberg F. Keeser D.. (2020). rTMS and tDCS for the treatment of catatonia: a systematic review. Schizophr. Res. 222, 73–78. 10.1016/j.schres.2020.05.028 32600779

[B81] Haroche A. Giraud N. Vinckier F. Amad A. Rogers J. Moyal M.. (2022). Efficacy of transcranial direct-current stimulation in catatonia: a review and case series. Front. Psychiatr. 13:876834. 10.3389/fpsyt.2022.876834 35573356 PMC9093033

[B82] Harris K. M. Fiala J. C. Ostroff L. (2003). Structural changes at dendritic spine synapses during long-term potentiation. Philos. Trans. R. Soc. Lond. B. Biol. Sci. 358, 745–748. 10.1098/rstb.2002.1254 12740121 PMC1693146

[B83] Hartwigsen G. Silvanto J. (2023). Noninvasive brain stimulation: multiple effects on cognition. Neuroscientist 29, 639–653. 10.1177/10738584221113806 35904354

[B84] Henley J. M. Wilkinson K. A. (2013). AMPA receptor trafficking and the mechanisms underlying synaptic plasticity and cognitive aging. Dialogues Clin. Neurosci. 15, 11–27. 10.31887/DCNS.2013.15.1/jhenley 23576886 PMC3622464

[B85] Hogan M. K. Hamilton G. F. Horner P. J. (2020). Neural stimulation and molecular mechanisms of plasticity and regeneration: a review. Front. Cell Neurosci.14:271. 10.3389/fncel.2020.00271 33173465 PMC7591397

[B86] Huang Y. J. Lee K. H. Grau J. W. (2017). Complete spinal cord injury (SCI) transforms how brain derived neurotrophic factor (BDNF) affects nociceptive sensitization. Exp. Neurol. 288, 38–50. 10.1016/j.expneurol.2016.11.001 27818188

[B87] Huang Y. J. Lee K. H. Murphy L. Garraway S. M. Grau J. W. (2016). Acute spinal cord injury (SCI) transforms how GABA affects nociceptive sensitization. Exp. Neurol. 285, 82–95. 10.1016/j.expneurol.2016.09.005 27639636 PMC5926208

[B88] Imamura K. Morii H. Nakadate K. Yamada T. Mataga N. Watanabe Y.. (2006). Brain-derived neurotrophic factor enhances expression of superior cervical ganglia clone 10 in lateral geniculate nucleus and visual cortex of developing kittens. Eur. J. Neurosci. 23, 637–648. 10.1111/j.1460-9568.2006.04592.x 16487145

[B89] Jacola L. M. Byars A. W. Hickey F. Vannest J. Holland S. K. Schapiro M. B.. (2014). Functional magnetic resonance imaging of story listening in adolescents and young adults with D own syndrome: evidence for atypical neurodevelopment. J. Intellect Disabil. Res. 58, 892–902. 10.1111/jir.12089 23962356

[B90] Jarrold C. Towse J. N. (2006). Individual differences in working memory. Neuroscience 139, 39–50. 10.1016/j.neuroscience.2005.07.002 16325344

[B91] Jones K. T. Stephens J. A. Alam M. Bikson M. Berryhill M. E. (2015). (2015). Longitudinal neurostimulation in older adults improves working memory. PLoS ONE 10:e0129751. 10.1371/journal.pone.0121904 25849358 PMC4388845

[B92] Jung D. H. Ahn S. M. Pak M. E. Lee H. J. Jung Y. J. Kim K. B.. (2020).Therapeutic effects of anodal transcranial direct current stimulation in a rat model of ADHD. Elife 21:e56359. 10.7554/eLife.56359.sa2 PMC7535928 32955434

[B93] Kaur G. Sharma A. Xu W. Gerum S. Alldred M. J. Subbanna S.. (2014). Glutamatergic transmission aberration: a major cause of behavioral deficits in a murine model of Down's syndrome. J Neurosci. (2014) 34, 5099–106. 10.1523/JNEUROSCI.5338-13.2014 24719089 PMC3983795

[B94] Kim S. Stephenson M. C. Morris P. G. Jackson S. R. (2014). tDCS-induced alterations in GABA concentration within primary motor cortex predict motor learning and motor memory: a 7 T magnetic resonance spectroscopy study. Neuroimage 99, 237–243. 10.1016/j.neuroimage.2014.05.070 24904994 PMC4121086

[B95] Kleschevnikov A. M. Yu J. Kim J. Lysenko L. V. Zeng Z. Yu Y. E.. (2017). Evidence that increased Kcnj6 gene dose is necessary for deficits in behavior and dentate gyrus synaptic plasticity in the Ts65Dn mouse model of Down syndrome. Neurobiol. Dis. 103, 1–10. 10.1016/j.nbd.2017.03.009 28342823 PMC5446050

[B96] Knudsen E. I. (2004). Sensitive periods in the development of the brain and behavior. J. Cogn. Neurosci.16, 1412–1425. 10.1162/0898929042304796 15509387

[B97] Kohlenberg T. M. Trelles M. P. McLarney B. Betancur C. Thurm A. Kolevzon A.. (2020). Psychiatric illness and regression in individuals with Phelan-McDermid syndrome. J. Neurodev. Disord. 12:7. 10.1186/s11689-020-9309-6 32050889 PMC7014655

[B98] Kolb B. Gibb R. (2011). Brain plasticity and behaviour in the developing brain. J. Can. Acad. Child. Adolesc. Psychiatr. 20, 265–276. 22114608 PMC3222570

[B99] Lanfranchi S. Jerman O. Dal Pont E. Alberti A. Vianello R. (2010). executive function in adolescents with down syndrome. J. Int. Disabil. Res. 54, 308–319. 10.1111/j.1365-2788.2010.01262.x 20202074

[B100] Lanfranchi S. Jerman O. Vianello R. (2009). Working memory and cognitive skills in individuals with down syndrome. Child Neuropsychol. 15, 397–416. 10.1080/09297040902740652 19274603

[B101] Lanfranchi S. Pulina F. Carretti B. Mammarella I. C. (2017). Training spatial-simultaneous working memory in individuals with Down syndrome. Res. Dev. Disabil. 64, 118–129. 10.1016/j.ridd.2017.03.012 28388504

[B102] Lang N. Siebner H. R. Ward N. S. Lee L. Nitsche M. A. Paulus W.. (2005). How does transcranial DC stimulation of the primary motor cortex alter regional neuronal activity in the human brain? Eur. J. Neurosci. 22, 495–504. 10.1111/j.1460-9568.2005.04233.x 16045502 PMC3717512

[B103] Lazzaro G. Bertoni S. Menghini D. Costanzo F. Franceschini S. Varuzza C.. (2021). Beyond reading modulation: temporo-parietal tDCS alters visuo-spatial attention and motion perception in dyslexia. Brain Sci. 19:263. 10.3390/brainsci11020263 33669651 PMC7922381

[B104] Lazzaro G. Fuc,à E. Caciolo C. Battisti A. Costanzo F. Varuzza C.. (2022). Understanding the effects of transcranial electrical stimulation in numerical cognition: a systematic review for clinical translation. J. Clin. Med. 11:2082. 10.3390/jcm11082082 35456176 PMC9032363

[B105] Lefaucheur J. P. Antal A. Ayache S. S. Benninger D. H. Brunelin J. Cogiamanian F.. (2017). Evidence-based guidelines on the therapeutic use of transcranial direct current stimulation (tDCS). Clin. Neurophysiol. 128, 56–92. 10.1016/j.clinph.2016.10.087 27866120

[B106] Leffa D. T. Bellaver B. Salvi A. A. de Oliveira C. Caumo W. Grevet E. H.. (2018). Transcranial direct current stimulation improves long-term memory deficits in an animal model of attention-deficit/hyperactivity disorder and modulates oxidative and inflammatory parameters. Brain Stimul. 11, 743–751. 10.1016/j.brs.2018.04.001 29656905

[B107] Liebetanz D. Nitsche M. A. Tergau F. Paulus W. (2002). Pharmacological approach to the mechanisms of transcranial DC-stimulation-induced after-effects of human motor cortex excitability. Brain 125, 2238–2247. 10.1093/brain/awf238 12244081

[B108] Lisman J. E. (2001). Three Ca^2+^ levels affect plasticity differently: the LTP zone, the LTD zone and no man's land. J. Physiol. 532:285. 10.1111/j.1469-7793.2001.0285f.x 11306649 PMC2278561

[B109] Longo V. Barbati S. A. Re A. Paciello F. Bolla M. Rinaudo M.. (2022). Transcranial direct current stimulation enhances neuroplasticity and accelerates motor recovery in a stroke mouse model. Stroke 53, 1746–1758. 10.1161/STROKEAHA.121.034200 35291824

[B110] Lopes J. Miziara I. Kahani D. Parreira R. Fonseca D. Lazzari R.. (2022). Brain wave behavior in children with down syndrome following cortical neuromodulation combined with sensorimotor stimulation: observational study. Physiother Theory Pract. 1–11. 10.1080/09593985.2022.2147808 36384401

[B111] Lopes J. B. P. Grecco L. A. C. Moura R. C. F. Lazzari R. D. Duarte N. A. C. Miziara I.. (2017). Protocol study for a randomised, controlled, double-blind, clinical trial involving virtual reality and anodal transcranial direct current stimulation for the improvement of upper limb motor function in children with Down syndrome. BMJ Open. 7:e016260. 10.1136/bmjopen-2017-016260 28801420 PMC5629662

[B112] Lopes J. B. P. Miziara I. M. Galli M. Cimolin V. Oliveira C. S. (2020). Effect of Transcranial direct current stimulation combined with Xbox-kinect game experience on upper limb movement in down syndrome: a case report. Front Bioeng Biotechnol. 8:514. 10.3389/fbioe.2020.00514 32548102 PMC7273846

[B113] Lopes J. B. P. Miziara I. M. Kahani D. Parreira R. B. de Almeida Carvalho Duarte N. Lazzari R. D.. (2022). Brain activity and upper limb movement analysis in children with Down syndrome undergoing transcranial direct current stimulation combined with virtual reality training: study protocol for a randomized controlled trial. Trials. 23:87. 10.1186/s13063-022-06014-4 35090554 PMC8796535

[B114] Lorenzon N. Musoles-Lleó J. Turrisi F. Gomis-González M. De La Torre R. Dierssen M.. (2023). State-of-the-art therapy for Down syndrome. Dev. Med. Child Neurol. 65, 870–884. 10.1111/dmcn.15517 36692980

[B115] Lu B. (2003). BDNF and activity-dependent synaptic modulation. Learn Mem.10, 86–98. 10.1101/lm.54603 12663747 PMC5479144

[B116] Maffei A. Charrier C. Caiati M. D. Barberis A. Mahadevan V. Woodin M. A.. (2017). Emerging mechanisms underlying dynamics of GABAergic synapses. J. Neurosci. 37, 10792–10799. 10.1523/JNEUROSCI.1824-17.2017 29118207 PMC5678011

[B117] Mahoney G. Wheeden C. A. Perales F. (2004). Relationship of preschool special education outcomes to instructional practices and parent-child interaction. Res. Dev. Disabil. 25, 539–558. 10.1016/j.ridd.2004.04.001 15541631

[B118] Mallei A. Baj G. Ieraci A. Corna S. Musazzi L. Lee F. S.. (2015). Expression and dendritic trafficking of BDNF-6 splice variant are impaired in knock-in mice carrying human BDNF Val66Met polymorphism. Int. J. Neuropsychopharmacol. 18:pyv069. 10.1093/ijnp/pyv069 26108221 PMC4675980

[B119] Málly J. (2013). Non-invasive brain stimulation (rTMS and tDCS) in patients with aphasia: mode of action at the cellular level. Brain Res. Bull. 98, 30–35. 10.1016/j.brainresbull.2013.07.005 23872450

[B120] Mancuso L. E. Ilieva I. P. Hamilton R. H. Farah M. J. (2016). Does transcranial direct current stimulation improve healthy working memory?: A meta-analytic review. J. Cognit. Neurosci. 28, 1063–1089. 10.1162/jocn_a_00956 27054400

[B121] Marangolo P. Fiori V. Gelfo F. Shofany J. Razzano C. Caltagirone C.. (2014). Bihemispheric tDCS enhances language recovery but does not alter BDNF levels in chronic aphasic patients. Restor. Neurol. Neurosci. 32, 367–379. 10.3233/RNN-130323 24398720

[B122] Marangolo P. Marinelli C. V. Bonifazi S. Fiori V. Ceravolo M. G. Provinciali L.. (2011). Electrical stimulation over the left inferior frontal gyrus (IFG) determines long-term effects in the recovery of speech apraxia in three chronic aphasics. Behav. Brain Res. 225, 498–504. 10.1016/j.bbr.2011.08.008 21856336

[B123] Martin D. M. Liu R. Alonzo A. Green M. Loo C. K. (2014). Use of transcranial direct current stimulation (tDCS) to enhance cognitive training: effect of timing of stimulation. Exp. Brain Res. 232, 3345–3351. 10.1007/s00221-014-4022-x 24992897

[B124] Martínez Cué C. Dierssen M. (2020). Plasticity as a therapeutic target for improving cognition and behavior in Down syndrome. Prog Brain Res. 251:269-302. 10.1016/bs.pbr.2019.11.001 32057310

[B125] Martínez-Cu,é C. Martínez P. Rueda N. Vidal R. García S. Vidal V.. (2013). Reducing GABAA α5 receptor-mediated inhibition rescues functional and neuromorphological deficits in a mouse model of down syndrome. J. Neurosci. 33, 3953–3966. 10.1523/JNEUROSCI.1203-12.2013 23447605 PMC6619314

[B126] Martínez-Cué C. Baamonde C. Lumbreras M. Paz J. Davisson M. T. Schmidt C.. (2002). Differential effects of environmental enrichment on behavior and learning of male and female Ts65Dn mice, a model for Down syndrome. Behav. Brain Res. 134, 185–200. 10.1016/S0166-4328(02)00026-8 12191805

[B127] Meinzer M. Darkow R. Lindenberg R. Flöel A. (2016). Electrical stimulation of the motor cortex enhances treatment outcome in post-stroke aphasia. Brain 139, 1152–1163. 10.1093/brain/aww002 26912641

[B128] Même S. Joudiou N. Yousfi N. Szeremeta F. Lopes-Pereira P. Beloeil J.. (2014). In vivo 9.4T MRI and 1H MRS for evaluation of brain structural and metabolic changes in the Ts65Dn mouse model for down syndrome. World J. Neurosci. 4, 152–163. 10.4236/wjns.2014.42018

[B129] Menghini D. Costanzo F. Vicari S. (2011). Relationship between brain and cognitive processes in Down syndrome. Behav. Genet. 41, 381–393. 10.1007/s10519-011-9448-3 21279430

[B130] Molosh A. I. Johnson P. L. Spence J. P. Arendt D. Federici L. M. Bernabe C.. (2014). Social learning and amygdala disruptions in Nf1 mice are rescued by blocking p21-activated kinase. Nat. Neurosci. 17, 1583–1590. 10.1038/nn.3822 25242307 PMC4213300

[B131] Moyal M. Plaze M. Baruchet A. Attali D. Cravero C. Raffin M.. (2022). Efficacity of tDCS in catatonic patients with Phelan McDermid syndrome, a case series. Brain Stimul. 15, 1432–1434. 10.1016/j.brs.2022.10.005 36309344

[B132] Nasseri P. Nitsche M. A. Ekhtiari H. (2015). A framework for categorizing electrode montages in transcranial direct current stimulation. Front. Hum. Neurosci. 9, 54. 10.3389/fnhum.2015.00054 25705188 PMC4319395

[B133] Neil N. Jones E. A. (2018). Communication intervention for individuals with Down syndrome: Systematic review and meta-analysis. Dev. Neurorehab. 21, 1–12. 10.1080/17518423.2016.1212947 27537068

[B134] Nissim N. R. Harvey D. Y. Haslam C. Friedman L. Bharne P. Litz G.. (2022). Through thick and thin: baseline cortical volume and thickness predict performance and response to transcranial direct current stimulation in primary progressive aphasia. Front. Hum. Neurosci. 16:907425. 10.3389/fnhum.2022.907425 35874157 PMC9302040

[B135] Nissim N. R. Moberg P. J. Hamilton R. H. (2020). Efficacy of noninvasive brain stimulation (tDCS or TMS) paired with language therapy in the treatment of primary progressive aphasia: an exploratory meta-analysis. Brain Sci. 10:597. 10.3390/brainsci10090597 32872344 PMC7563447

[B136] Nitsche M. A. Fricke K. Henschke U. Schlitterlau A. Liebetanz D. Lang N.. (2003). Pharmacological modulation of cortical excitability shifts induced by transcranial direct current stimulation in humans. J. Physiol. 15, 293–301. 10.1113/jphysiol.2003.049916 12949224 PMC2343495

[B137] Nitsche M. A. Paulus W. (2000). Excitability changes induced in the human motor cortex by weak transcranial direct current stimulation. J. Physiol. 527, 633–639. 10.1111/j.1469-7793.2000.t01-1-00633.x 10990547 PMC2270099

[B138] Nitsche M. A. Paulus W. (2001). Sustained excitability elevations induced by transcranial DC motor cortex stimulation in humans. Neurology 57, 1899–1901. 10.1212/WNL.57.10.1899 11723286

[B139] O'Toole C. Chiat S. (2006). Symbolic functioning and language development in children with Down syndrome. Intl. J. Lang. Comm. Disor. 41, 155–171. 10.1080/13682820500221600 16546893

[B140] Pelletier S. J. Lagacé M. St-Amour I. Arsenault D. Cisbani G. Chabrat A.. (2014). The morphological and molecular changes of brain cells exposed to direct current electric field stimulation. Int J Neuropsychopharmacol. 7:pyu090. 10.1093/ijnp/pyu090 25522422 PMC4376545

[B141] Pennington B. F. Moon J. Edgin J. Stedron J. Nadel L. (2003). The neuropsychology of Down syndrome: evidence for hippocampal dysfunction. Child Dev. 74, 75–93. 10.1111/1467-8624.00522 12625437

[B142] Philippen S. Hanert A. Schönfeld R. Granert O. Yilmaz R. Jensen-Kondering U.. (2024). Transcranial direct current stimulation of the right temporoparietal junction facilitates hippocampal spatial learning in Alzheimer's disease and mild cognitive impairment. Clin. Neurophysiol. 157, 48–60. 10.1016/j.clinph.2023.11.003 38056370

[B143] Podda M. V. Cocco S. Mastrodonato A. Fusco S. Leone L. Barbati S. A.. (2016). Anodal transcranial direct current stimulation boosts synaptic plasticity and memory in mice via epigenetic regulation of Bdnf expression. Sci. Rep. 6:22180. 10.1038/srep22180 26908001 PMC4764914

[B144] Poje A. B. Manzardo A. Gustafson K. M. Liao K. Martin L. E. Butler M. G. (2021). Effects of Transcranial Direct Current Stimulation (tDCS) on Go/NoGo performance using food and non-food stimuli in patients with prader-willi syndrome. Brain Sci. 11:250. 10.3390/brainsci11020250 33671295 PMC7922059

[B145] Pujol J. Del Hoyo L. Blanco-Hinojo L. De Sola S. Macià D. Martínez-Vilavella G.. (2015). Anomalous brain functional connectivity contributing to poor adaptive behavior in Down syndrome. Cortex 64, 148–156. 10.1016/j.cortex.2014.10.012 25461715

[B146] Pulina F. Carretti B. Lanfranchi S. Mammarella I. C. (2015). Improving spatial-simultaneous working memory in Down syndrome: effect of a training program led by parents instead of an expert. Front. Psychol. 6:1265. 10.3389/fpsyg.2015.01265 26379590 PMC4547001

[B147] Puri R. Hinder M. R. Fujiyama H. Gomez R. Carson R. G. Summers J. J.. (2015). Duration-dependent effects of the BDNF Val66Met polymorphism on anodal tDCS induced motor cortex plasticity in older adults: a group and individual perspective. Front. Aging Neurosci. 7:107. 10.3389/fnagi.2015.00107 26097454 PMC4456583

[B148] Raveendran V. A. Pressey J. C. Woodin M. A. (2020). A novel small molecule targets NKCC1 to restore synaptic inhibition. Trends Pharmacol. Sci. 41, 897–899. 10.1016/j.tips.2020.10.002 33097285

[B149] Reinhart R. M. Cosman J. D. Fukuda K. Woodman G. F. (2017). Using transcranial direct-current stimulation (tDCS) to understand cognitive processing. Atten. Percept. Psychophys. 79, 3–23. 10.3758/s13414-016-1224-2 27804033 PMC5539401

[B150] Reynolds Losin E. A. Rivera S. M. O'Hare E. D. Sowell E. R. Pinter J. D. (2009). Abnormal fMRI activation pattern during story listening in individuals with down syndrome. Am. J. Int. Dev. Disab. 114, 369–380. 10.1352/1944-7558-114.5.369 19928018

[B151] Rigoldi C. Galli M. Albertini G. (2011). Gait development during lifespan in subjects with down syndrome. Res. Dev. Disabil. 32, 158–163. 10.1016/j.ridd.2010.09.009 20943345

[B152] Rodenbusch T. L. M. Ribeiro T. S. Simão C. R. Britto H. M. J. S. Tudella E. Lindquist A. R.. (2013). Effects of treadmill inclination on the gait of children with Down syndrome. Res. Dev. Disabil. 34, 2185–2190. 10.1016/j.ridd.2013.02.014 23643771

[B153] Rohan J. G. Carhuatanta K. A. McInturf S. M. Miklasevich M. K. Jankord R. (2015). Modulating hippocampal plasticity with in vivo brain stimulation. J. Neurosci. 16, 12824–12832. 10.1523/JNEUROSCI.2376-15.2015 26377469 PMC4643097

[B154] Romei V. Thut G. Silvanto J. (2016). Information-based approaches of noninvasive transcranial brain stimulation. Trends Neurosci. 39, 782–795. 10.1016/j.tins.2016.09.001 27697295

[B155] Ruiz-Mejias M. Martinez de Lagran M. Mattia M. Castano-Prat P. Perez-Mendez L. Ciria-Suarez L. (2016). Overexpression of Dyrk1A, a down syndrome candidate, decreases excitability and impairs gamma oscillations in the prefrontal cortex. J. Neurosci. 36, 3648–3659. 10.1523/JNEUROSCI.2517-15.2016 27030752 PMC6601739

[B156] Rvachew S. Folden M. (2018). Speech therapy in adolescents with Down syndrome: in pursuit of communication as a fundamental human right. Int. J. Speech Lang. Pathol. 20, 75–83. 10.1080/17549507.2018.1392605 29124959

[B157] Salehinejad M. A. Ghanavati E. Glinski B. Hallajian A. H. Azarkolah A. (2022). A systematic review of randomized controlled trials on efficacy and safety of transcranial direct current stimulation in major neurodevelopmental disorders: ADHD, autism, and dyslexia. Brain Behav.12:e2724. 10.1002/brb3.2724 35938945 PMC9480913

[B158] Sánchez-León C. A. Cordones I. Ammann C. Ausín J. M. Gómez-Climent M. A. Carretero-Guillén A.. (2021). Immediate and after effects of transcranial direct-current stimulation in the mouse primary somatosensory cortex. Sci. Rep. 4, 3123. 10.1038/s41598-021-82364-4 33542338 PMC7862679

[B159] Santin M. D. Valabrègue R. Rivals I. Pénager R. Paquin R. Dauphinot L.. (2014). In vivo 1H MRS study in microlitre voxels in the hippocampus of a mouse model of Down syndrome at 11, 7. T. NMR Biomed. 27, 1143–1150. 10.1002/nbm.3155 25088227

[B160] Santoro S. L. Cannon S. Capone G. Franklin C. Hart S. J. Hobensack V.. (2020). Unexplained regression in Down syndrome: 35 cases from an international Down syndrome database. Genet. Med. 22, 767–776. 10.1038/s41436-019-0706-8 31767984

[B161] Santos C. A. Franco de Moura R. C. Lazzari R. D. Dumont A. J. Braun L. A. Oliveira C. S. (2015). Upper limb function evaluation scales for individuals with cerebral palsy: a systematic review. J. Phys. Ther. Sci. 27, 1617–1620. 10.1589/jpts.27.1617 26157275 PMC4483453

[B162] Savardi A. Patricelli Malizia A. De Vivo M. Cancedda L. Borgogno M. (2023). Preclinical development of the Na-K-2Cl Co-transporter-1 (NKCC1) inhibitor ARN23746 for the treatment of neurodevelopmental disorders. ACS Pharmacol. Transl. Sci. 6, 1–11. 10.1021/acsptsci.2c00197 36654749 PMC9841778

[B163] Schneider H. D. Hopp J. P. (2011). The use of the Bilingual Aphasia Test for assessment and transcranial direct current stimulation to modulate language acquisition in minimally verbal children with autism. Clin. Linguist. Phon. 25, 640–654. 10.3109/02699206.2011.570852 21631313

[B164] Seager E. Sampson S. Sin J. Pagnamenta E. Stojanovik V. (2022). A systematic review of speech, language and communication interventions for children with Down syndrome from 0 to 6 years. Intl. J. Lang. Comm. Disor. 57, 441–463. 10.1111/1460-6984.12699 35191587

[B165] Smith E. Hokstad S. Næss K.-.A. B. (2020). Children with Down syndrome can benefit from language interventions; Results from a systematic review and meta-analysis. J. Commun. Disorders 85:105992. 10.1016/j.jcomdis.2020.105992 32445828

[B166] Sousa B. Martins J. Castelo-Branco M. Gonçalves J. (2022). transcranial direct current stimulation as an approach to mitigate neurodevelopmental disorders affecting excitation/inhibition balance: focus on autism spectrum disorder, schizophrenia, and attention deficit/hyperactivity disorder. J. Clin. Med. 18:2839. 10.3390/jcm11102839 35628965 PMC9143428

[B167] Stagg C. J. Best J. G. Stephenson M. C. O'Shea J. Wylezinska M. Kincses Z. T.. (2009). Polarity-sensitive modulation of cortical neurotransmitters by transcranial stimulation. J. Neurosci. 22, 5202–5206. 10.1523/JNEUROSCI.4432-08.2009 19386916 PMC6665468

[B168] Stagg C. J. Nitsche M. A. (2011). Physiological basis of transcranial direct current stimulation. Neuroscientist 17, 37–53. 10.1177/1073858410386614 21343407

[B169] Strube W. Nitsche M. A. Wobrock T. Bunse T. Rein B. Herrmann M.. (2014). BDNF-Val66Met-polymorphism impact on cortical plasticity in schizophrenia patients: a proof-of-concept study. Int. J. Neuropsychopharmacol. 31, pyu040. 10.1093/ijnp/pyu040 25612896 PMC4360229

[B170] Teo F. Hoy K. E. Daskalakis Z. J. Fitzgerald P. B. (2011). Investigating the role of current strength in tDCS modulation of working memory performance in healthy controls. Front. Psychiatry 2:45. 10.3389/fpsyt.2011.00045 21811474 PMC3141358

[B171] Vacca R. A. Augello A. Gallo L. Caggianese G. Malizia V. La Grutta S.. (2023). Serious Games in the new era of digital-health interventions: a narrative review of their therapeutic applications to manage neurobehavior in neurodevelopmental disorders. Neurosci. Biobehav. Rev. 149:105156. 10.1016/j.neubiorev.2023.105156 37019246

[B172] van der Vliet R. Jonker Z. D. Louwen S. C. Heuvelman M. de Vreede L. Ribbers G. M.. (2018). Cerebellar transcranial direct current stimulation interacts with BDNF Val66Met in motor learning. Brain Stimul. 11, 759–771. 10.1016/j.brs.2018.04.009 29680227

[B173] Van Steenburgh J. J. Varvaris M. Schretlen D. J. Vannorsdall T. D. Gordon B. (2017). Balanced bifrontal transcranial direct current stimulation enhances working memory in adults with high-functioning autism: a sham-controlled crossover study. Mol. Autism. 28:40. 10.1186/s13229-017-0152-x 28775825 PMC5534041

[B174] Vega J. N. Hohman T. J. Pryweller J. R. Dykens E. M. Thornton-Wells T. A. (2015). Resting-state functional connectivity in individuals with Down syndrome and Williams syndrome compared with typically developing controls. Brain Connect. 5, 461–475. 10.1089/brain.2014.0266 25712025 PMC4601631

[B175] Velikova S. Magnani G. Arcari C. Falautano M. Franceschi M. Comi G.. (2011). Cognitive impairment and EEG background activity in adults with Down's syndrome: a topographic study. Hum. Brain Mapp. 32, 716–729. 10.1002/hbm.21061 21484947 PMC6870078

[B176] Wang J. Tian J. Hao R. Tian L. Liu Q. (2018). Transcranial direct current stimulation over the right DLPFC selectively modulates subprocesses in working memory. PeerJ. 6:e4906. 10.7717/peerj.4906 29868292 PMC5978386

[B177] White N. S. Alkire M. T. Haier R. J. (2003). A voxel-based morphometric study of nondemented adults with Down Syndrome. NeuroImage 20, 393–403. 10.1016/S1053-8119(03)00273-8 14527599

[B178] Wiegand A. Nieratschker V. Plewnia C. (2016). Genetic modulation of transcranial direct current stimulation effects on cognition. Front. Hum. Neurosci. 10:651. 10.3389/fnhum.2016.00651 28066217 PMC5177633

[B179] Wilkinson K. M. Finestack L. H. (2020). Multimodal AAC for Individuals With Down Syndrome, 1st Edn. Baltimore: Paul H. Brookes Publishing Co.

[B180] Wirth M. Rahman R. A. Kuenecke J. Koenig T. Horn H. Sommer W.. (2011). Effects of transcranial direct current stimulation (tDCS) on behaviour and electrophysiology of language production. Neuropsychologia. 49, 3989–3998. 10.1016/j.neuropsychologia.2011.10.015 22044650

[B181] Woodward J. (2010). Causation in biology: stability, specificity, and the choice of levels of explanation. Biol Philos 25, 287–318. 10.1007/s10539-010-9200-z

[B182] Yu T. H. Wu Y. J. Chien M. E. Hsu K. S. (2019). Transcranial direct current stimulation induces hippocampal metaplasticity mediated by brain-derived neurotrophic factor. Neuropharmacology 144, 358–367. 10.1016/j.neuropharm.2018.11.012 30439417

[B183] Zanos S. Richardson A. G. Shupe L. Miles F. P. Fetz E. E. (2011). The Neurochip-2: an autonomous head-fixed computer for recording and stimulating in freely behaving monkeys. IEEE Trans. Neural. Syst. Rehabil. Eng. 19, 427–435. 10.1109/TNSRE.2011.2158007 21632309 PMC3159515

[B184] Zettin M. Bondesan C. Nada G. Varini M. Dimitri D. (2021). Transcranial direct-current stimulation and behavioral training, a promising tool for a tailor-made post-stroke aphasia rehabilitation: a review. Front. Hum. Neurosci. 15:742136. 10.3389/fnhum.2021.742136 34987366 PMC8722401

[B185] Zhao X. Ding J. Pan H. Zhang S. Pan D. Yu H.. (2020). Anodal and cathodal tDCS modulate neural activity and selectively affect GABA and glutamate syntheses in the visual cortex of cats. J. Physiol. 598, 3727–3745. 10.1113/JP279340 32506434

