# Supplementary material for: Transcranial Direct Current Stimulation in neurogenetic syndromes: new treatment perspectives for Down syndrome?
Source: Front Cell Neurosci. 2024 Feb 22;18:1328963. doi: 10.3389/fncel.2024.1328963 (PMC10917937; doi:10.3389/fncel.2024.1328963)
Supplement: Supplementary file 1 [file Table_1.pdf]

Supplementary

1. tDCS application in neurogenetic syndromes

A growing body of human and some of animal studies and indicates that the use of transcranial direct current stimulation (tDCS) may induce behavioural improvements in individuals with neurodevelopmental disorders, such as autism spectrum disorder (ASD), attention deficit/hyperactivity disorder (ADHD) and learning disorders (Schneider and Hopp, 2011; Costanzo et al., 2016; Van Steenburgh et al., 2017; Lazzaro et al., 2021; Esse Wilson et al., 2018; Leffa et al., 2018; Hadar et al., 2020; Jung et al., 2020; Han et al., 2022; Lazzaro et al., 2022; Salehinejad et al., 2022; Sousa et al., 2022; Chen et al., 2023).
In order to report relevant recent studies on the therapeutic potential of tDCS, we considered the current state of the application of tDCS in five neurogenetic disorders: Rett Syndrome (RTT), Prader-Willi Syndrome (PWS), Neurofibromatosis type 1 syndrome (NF1), Phelan-McDermid syndrome (PMS), and Down Syndrome (DS). The following paragraphs summarize detailed results of the published studies on RTT, PWS, NF1 and PMS.

Rett syndrome

Mutations in the X-linked gene encoding a regulator of epigenetic gene expression, methyl CpG binding protein (MeCP2), cause complex genetic disorder like RTT (Amir et al., 1999; Amir et al., 2000). RTT syndrome was described for the first time in 1966 by Andreas Rett (Rett A., 1966) and it affects approximately 1 in 10,000 to 15,000 females (Hagberg and Hagberg, 1997). Individuals with RTT usually exhibit normal development during the first 6 to 18 months of life, followed by severe developmental delay with a rapid regression characterized by a lose acquired speech, hand -use, slow cranial growth, seizures, autistic features, ataxia, gait apraxia (Amir et al., 2000). Compelling evidence supports a widespread dendritic and synaptic disorder (Kaufmann and Moser, 2000; Kaufmann et al., 2016). Moreover, it has been suggested that crucial aspects lie in the balance and spatiotemporal connection between excitation and inhibition, rather than isolated deficits in either excitation or inhibition (Banerjee et al., 2019). Fabio and colleagues designed a study to examine the neurophysiological and cognitive effects of tDCS combined with a training program in three girls with RTT exhibiting chronic language impairments (Fabio et al., 2018). In particular, the authors explored for the first time a combined approach consisting of a training program based on cognitive-behavioural strategies, such as imitation procedures, prompting, generalization, combined with tDCS on Broca's area; a general enhancement in language abilities has been reported. A subsequent work from the same authors applied tDCS combined with traditional rehabilitative techniques such as cognitive–behavioral strategies, to examine their effects on attention and language in participants with RTT (Fabio et al., 2020). The study included thirty-five female young adults randomly allocated into two groups: active tDCS and sham tDCS, both receiving cognitive empowerment for the production of vowels, phonemes and words during the treatment phase. They applied the anode electrode over the primary motor cortex and the cathode electrode over the right supraorbital region. All participants received tDCS treatment plus cognitive empowerment for 10 daily sessions over a 1-week period, resulting in an improvement in attention and language in individuals with RTT.

Prader-Willi syndrome

PWS is a rare neurodevelopmental genetic disorder with an incidence of about one in 10,000–20,000 live births and is characterized by Intellectual Disability (ID) and insatiable appetite, hypotonia multiple system, growth hormone deficiencies, hyperphagia leading to severe obesity with detrimental health consequences. Moreover, the syndrome is characterized by physical and cognitive characteristics such as short stature, small hands and feet and mild cognitive problems and behavioural disturbances. Evidence shows abnormal activity or morphology of the neural networks that involves the dorsolateral prefrontal cortex (DLPFC) and correlated area, such as the hypothalamus, ventromedial prefrontal cortex, insula, and amygdala; the involvement of these areas is thought to contribute to food-intake decisions (Holsen et al., 2011; Zhang et al., 2015). A study of the hypothalamus in individuals with PWS revealed a transcriptomic signature marked by neuronal loss, disrupted neuroplasticity, and neuroinflammation (Bochukova et al., 2018). To date, robust clinical and preclinical evidence are not present in the literature evaluating the role of tDCS on neuroplasticity in individuals with PWS. However, the effects of tDCS have been evaluated clinically. A pilot double blind, sham-controlled, multicentre study of tDCS modulation (right DLPFC for anode/left supraorbital area for cathode) of food drive and craving in adult obese participants, participants with PWS, and adult healthy-weight controls, was conducted (Bravo et al., 2016). In participants with PWS the effect of consecutive daily sessions of active or sham tDCS over the right DLPFC was evaluated. Their findings support sustained neuromodulatory effects and efficacy of tDCS to reduce food drive and behaviours affecting hyperphagia in PWS. Given the limited therapeutic options for PWS, tDCS has been applied to ameliorate hyperphagia, food craving, and aberrant behaviours in individuals with PWS. The hypothesis for its use is that modulation of prefrontal activity, intrinsically associated with food craving, might lead to control of this behavior (Boggio et al., 2009). Azevedo and colleagues observed amelioration of hyperphagic and food craving symptoms in adolescents and adults with PWS after sessions of tDCS, with the anode positioned over the left DLPFC, and cathode over the contralateral region (Azevedo et al., 2017, 2021). Another study showed the effects of tDCS on Go/NoGo performance using food and non-food stimuli in patients with PWS (Poje et al., 2021) using tDCS with the electrode placed in the right DLPFC for anode and left prefrontal cortex for cathode electrode. Results showed modulatory effect on EEG parameters during the NoGo condition and marginally significant effects in the task performance.

Neurofibromatosis type 1 syndrome

NF1 is a single gene disorder with a prevalence approximately of 1:2500 to 1:3500 individuals worldwide (Anderson and Gutmann, 2015). NF1 is associated with cutaneous manifestations, such as freckling on flexural areas and neurofibromas which are readily apparent on visual inspection and are usually the first sign of the disease (Miraglia et al., 2020), as well as higher risk of much rarer physical complications that could include disfiguring plexiform neurofibromas, scoliosis, pseudarthrosis, and optic nerve gliomas (Ferner et al., 2007). Despite Intelligence Quotient (IQ) being usually within the normal range, NF1 is associated with generalized neuropsychological impairment, involving academic skills, visuospatial skills, social competence, attention (Lehtonen et al., 2012), working memory (WM) (Pobric et al., 2021) and other executive functions (Plasschaert et al. 2016). Moreover, children with NF1 may exhibit behavioural problems, such as ADHD symptomatology, causing great parental distress (Garg et al., 2013). NF1 is characterized by a GABAergic overactivity and impairment in

synaptic plasticity (Costa et al., 2002; Cui et al., 2008; Molosh et al., 2014) due to the pathogenic variants of the NF1 gene that cause an increase in RasMAPKinase signaling. Animal models of NF1 showed that the learning deficits of *Nf1*+/− mice (Nf1 heterozygous null mutants; Nf1(+/-)) are due to increased activation of ERK signalling (Cui et al., 2008), with consequent higher levels of synapsin I phosphorylation, greater GABA release and deficits in long term potentiation (LTP) and learning (Shilyansk et al., 2010). Significant left DLPFC and right parietal cortex hypoactivation has been found in humans with NF1 through functional neuroimaging and electrophysiological studies during WM tasks (Ibrahim et al., 2017; Pobric et al., 2021).

Interestingly, a recent work by Garg and colleagues (Garg et al., 2022) investigated the responsiveness of the GABAergic system to the application of anodal tDCS to DLPFC in adolescents with NF1 with a focus on WM performance. The authors hypothesized that the administration of anodal tDCS to the left DLPFC would reduce GABA and improve performance on WM tasks, increasing brain activation in the targeted DLPFC. They found a greater reduction in GABA+ in comparison with the sham condition after anodal tDCS application, without effects on glutamate + glutamine, suggesting that tDCS modulated inhibitory activity in the DLPFC. However, no effects in WM performance were observed. In conclusion, they utilized the neurodevelopmental condition NF1 as a model to investigate the relationship between a cognitive function such as WM performance and inter-individual variations in GABA function (Garg et al., 2022).

Phelan–McDermid syndrome

PMS, also known as 22q13.3 deletion syndrome, is a neurodevelopmental disorder first described in 1985. It is characterized by developmental and severe speech delay, impairment in motor tone and function, and is considered to be a relatively common cause of ASD and ID, accounting for between 0.5 % and 2.0 % of cases (Watt et al., 1985, Costales and Kolevzo, 2015). Regarding the behavioural phenotype, frequently reported symptoms include hyperactivity, impulsiveness, restlessness, repetitive behaviour, sleep problems, and autistic like symptoms (Phelan and McDermid, 2011; Sarasua et al., 2014). The cause of PMS has been isolated to the loss of function of one copy of SH3 and multiple ankyrin repeat domains protein 3 (SHANK3), which codes for a master scaffolding protein found in the postsynaptic density of excitatory synapses. A reduction of SHANK3 expression leads to a decrease in the numbers of dendrites and impaired synaptic transmission and plasticity (Phelan et al., 1993). Groves Kuhnle and colleagues (2022) explored a mouse model deficient in SHANK3, the synaptic protein that is mutated in PMS and is often comorbid with ASD. SHANK3 knock-out mice exhibited abnormalities in homeostatic synaptic scaling (Groves Kuhnle et al., 2022). In the literature, there is only a case-series report that evaluated the effects of tDCS in individuals with PMS (Moyal et al., 2022). The authors described the efficacy and safety of tDCS in four young adults with PMS and co-occurring catatonia. In particular, participants received tDCS treatment with anode over the DLPFC and cathode over the left temporo-parietal junction. This protocol was previously found effective for schizophrenia and bipolar patients with catatonia (Haroche et al., 2022). After tDCS application, Moyal and colleagues observed an improvement in spontaneous speech in all cases with PMS, in one case, the individual was able to speak again after months of mutism. Additionally, all patients who received tDCS showed reduction of catatonic symptoms, experiencing tranquilization and a significant decrease in impulsivity.

2. References

Amir R.E., Van den Veyver I.B., Schultz R., Malicki D.M., Tran C.Q., Dahle E.J., Philippi A., Timar L., Percy A.K., Motil K.J., Lichtarge O., Smith E.O., Glaze D.G., Zoghbi H.Y. (2000). Influence of mutation type and X chromosome inactivation on Rett syndrome phenotypes. Ann Neurol. 47(5):670-9

Amir R.E., Van den Veyver I.B., Wan M., Tran C.Q., Francke U., Zoghbi H.Y. (1999). Rett syndrome is caused by mutations in X-linked MECP2, encoding methyl-CpG-binding protein 2. Nat Genet. 23(2):185-8. doi: 10.1038/13810

Anagnostopoulou A., Styliadis C., Kartsidis P., Romanopoulou E., Zilidou V., Karali C., Karagianni M., Klados M., Paraskevopoulos E., Bamidis P.D. (2021). Computerized physical and cognitive training improves the functional architecture of the brain in adults with Down syndrome: A network science EEG study. Netw Neurosci. 5(1):274-294. doi: 10.1162/netn_a_00177

Anderson J.S., Nielsen J.A., Ferguson M.A., Burback M.C., Cox E.T., Dai L., Gerig G., Edgin J.O., Korenberg J.R. (2013). Abnormal brain synchrony in Down Syndrome. Neuroimage Clin. 2:703-15. doi: 10.1016/j.nicl.2013.05.006

Azevedo C., Gomes J.S., Trevizol A.P., Dias Á.M., Cordeiro Q. (2017). At-Home Transcranial Direct Current Stimulation in Prader-Willi Syndrome With Severe Intellectual Disability: A Case Study. J ECT.33(3):e29-e30. doi: 10.1097/YCT.0000000000000409.

Azevedo, C.C., Trevizol, A.P., Gomes, J.S., Akiba, H., Franco, R.R., Simurro, P.B., Ianni, R.M., Grigolon, R.B., Blumberger, D.M., Dias, A.M. (2021). Transcranial Direct Current Stimulation for Prader-Willi Syndrome. J ECT. 37(1):58-63. doi: 10.1097/YCT.0000000000000722.

Banerjee A., Rikhye R.V., Breton-Provencher V., Tang X., Li C., Li K., Runyan C.A., Fu Z., Jaenisch R., Sur M. (2016). Jointly reduced inhibition and excitation underlies circuit-wide changes in cortical processing in Rett syndrome. Proc Natl Acad Sci U S A. 113(46):E7287-E7296. doi: 10.1073/pnas.1615330113.

Bochukova E.G., Lawler K., Croizier S., Keogh J.M., Patel N., Strohbehn G., Lo K.K., Humphrey J., Hokken-Koelega A., Damen L., Donze S., Bouret S.G., Plagnol V., Farooqi I.S. (2018). A Transcriptomic Signature of the Hypothalamic Response to Fasting and BDNF Deficiency in Prader-Willi Syndrome. Cell Rep. 22(13):3401-3408. doi: 10.1016/j.celrep.2018.03.018.

Boggio P.S., de Macedo E.C., Schwartzman J.S., Brunoni D., Teixeira M.C., Fregni F. (2009). Transcranial direct current stimulation: a novel approach to control hyperphagia in Prader-Willi syndrome. J Child Neurol. 24(5):642-3. doi: 10.1177/0883073808322339

Bravo G.L., Poje A.B., Perissinotti I., Marcondes B.F., Villamar M.F., Manzardo A.M., Luque L., LePage J.F., Stafford D., Fregni F., Butler M.G. (2016). Transcranial direct current stimulation reduces food-craving and measures of hyperphagia behavior in participants with Prader-Willi syndrome. Am J Med Genet B Neuropsychiatr Genet. 171B(2):266-75. doi: 10.1002/ajmg.b.32401

Carlesimo G.A., Marotta L., Vicari S. (1997). Long-term memory in mental retardation: evidence for a specific impairment in subjects with Down's syndrome. Neuropsychologia. 35(1):71-9. doi: 10.1016/s0028-3932(96)00055-3

Chen H.Y., Yang C.Y., Hsieh T.H., Peng C.W., Chuang L.L., Chang Y.L., Chi H.J., Lee H.M., Liang S.H. (2023). Effects of transcranial direct current stimulation on improving performance of delayed- reinforcement attentional set-shifting tasks in attention-deficit/hyperactivity disorder rat model. Behav Brain Res. 2;437:114145. doi: 10.1016/j.bbr.2022.114145

Costa R.M., Federov N.B., Kogan J.H., Murphy G.G., Stern J., Ohno M., Kucherlapati R., Jacks T., Silva A.J. (2002). Mechanism for the learning deficits in a mouse model of neurofibromatosis type 1. Nature. 415(6871):526-30. doi: 10.1038/nature711

Costales J.L., Kolevzon A. (2015). Phelan-McDermid Syndrome and SHANK3: Implications for Treatment. Neurotherapeutics. 12(3):620-30. doi: 10.1007/s13311-015-0352-z

Costanzo F., Varuzza C., Rossi S., Sdoia S., Varvara P., Oliveri M., Koch G., Vicari S., Menghini D. (2016). Reading changes in children and adolescents with dyslexia after transcranial direct current stimulation. Neuroreport. 23;27(5):295-300. doi: 10.1097/WNR.0000000000000536

Cramer N., Galdzicki Z. (2012). From abnormal hippocampal synaptic plasticity in down syndrome mouse models to cognitive disability in down syndrome. Neural Plast. 2012:101542. doi: 10.1155/2012/101542

Cui Y., Costa R.M., Murphy G.G., Elgersma Y., Zhu Y., Gutmann D.H., Parada L.F., Mody I., Silva A.J. (2008). Neurofibromin regulation of ERK signaling modulates GABA release and learning. Cell. 135(3):549-60. doi: 10.1016/j.cell.2008.09.060

Dierssen M., Benavides-Piccione R., Martínez-Cué C., Estivill X., Flórez J., Elston G.N., DeFelipe J. (2003). Alterations of neocortical pyramidal cell phenotype in the Ts65Dn mouse model of Down syndrome: effects of environmental enrichment. Cereb Cortex. 13(7):758-64. doi: 10.1093/cercor/13.7.758

Esse Wilson J., Trumbo M.C., Wilson J.K., Tesche C.D. (2018). Transcranial direct current stimulation (tDCS) over right temporoparietal junction (rTPJ) for social cognition and social skills in adults with autism spectrum disorder (ASD). J Neural Transm (Vienna). 125(12):1857-1866. doi: 10.1007/s00702-018-1938-5

Fabio R.A., Gangemi A., Capri T., Budden S., Falzone A. (2018). Neurophysiological and cognitive effects of Transcranial Direct Current Stimulation in three girls with Rett Syndrome with chronic language impairments. Res Dev Disabil. 76:76-87. doi: 10.1016/j.ridd.2018.03.008

Fabio R.A., Gangemi A., Semino M., Vignoli A., Canevini M.P., Priori A., Rosa G.D., Caprì T. (2020). Effects of Combined Transcranial Direct Current Stimulation with Cognitive Training in Girls with Rett Syndrome. Brain Sci. 10(5):276. doi: 10.3390/brainsci10050276

Ferner R.E., Huson S.M., Thomas N., Moss C., Willshaw H., Evans D.G., Upadhyaya M., Towers R., Gleeson M., Steiger C., Kirby A. (2007). Guidelines for the diagnosis and management of individuals with neurofibromatosis 1. J Med Genet. 44(2):81-8. doi: 10.1136/jmg.2006.045906

Garg S., Green J., Leadbitter K., Emsley R., Lehtonen A., Evans D.G., Huson S.M. (2013). Neurofibromatosis type 1 and autism spectrum disorder. Pediatrics. 132(6):e1642-8. doi: 10.1542/peds.2013-1868

Garg, S., Williams, S., Jung, J., Pobric, G., Nandi, T., Lim, B., Vassallo, G., Green, J., Evans, D.G., Stagg, C.J., Parkes, L.M., Stivaros, S. (2022). Non-invasive brain stimulation modulates GABAergic activity in neurofibromatosis 1. Sci Rep. 12(1):18297. doi: 10.1038/s41598-022-21907-9.

Groves Kuhnle C., Grimes M., Suárez Casanova V.M., Turrigiano G.G., Van Hooser S.D. (2022). Juvenile Shank3 KO Mice Adopt Distinct Hunting Strategies during Prey Capture Learning. eNeuro. 9(6):ENEURO.0230-22.2022. doi: 10.1523/ENEURO.0230-22.2022

Hadar R., Winter R., Edemann-Callesen H., Wieske F., Habelt B., Khadka N., Felgel-Farnholz V., Barroeta-Hlusicka E., Reis J., Tatarau C.A., Funke K., Fritsch B., Bernhardt N., Bikson M., Nitsche M.A., Winter C. (2020). Prevention of schizophrenia deficits via non-invasive adolescent frontal cortex stimulation in rats. Mol Psychiatry. 25(4):896-905. doi: 10.1038/s41380-019-0356-x

Hagberg B., Hagberg G. (1997). Rett syndrome: epidemiology and geographical variability. Eur Child Adolesc Psychiatry. 6 Suppl 1:5-7

Han Y.M.Y., Chan M.M.Y., Shea C.K.S., Lai O.L., Krishnamurthy K., Cheung M.C., Chan A.S. (2022). Neurophysiological and behavioral effects of multisession prefrontal tDCS and concurrent cognitive remediation training in patients with autism spectrum disorder (ASD): A double-blind, randomized controlled fNIRS study. Brain Stimul. 15(2):414-425. doi: 10.1016/j.brs.2022.02.004.

Haroche A., Giraud N., Vinckier F., Amad A., Rogers J., Moyal M., Canivet L., Berkovitch L., Gaillard R., Attali D., Plaze M. (2022). Efficacy of Transcranial Direct-Current Stimulation in Catatonia: A Review and Case Series. Front Psychiatry. 13:876834. doi: 10.3389/fpsyt.2022.876834.

Holsen L.M., Savage C.R., Martin L.E., Bruce A.S., Lepping R.J., Ko E., Brooks W.M., Butler M.G., Zarcone J.R., Goldstein J.M. (2011). Importance of reward and prefrontal circuitry in hunger and satiety: Prader-Willi syndrome vs simple obesity. Int J Obes (Lond). 36(5):638-47. doi: 10.1038/ijo.2011.204

Ibrahim A.F.A., Montojo C.A., Haut K.M., Karlsgodt K.H., Hansen L., Congdon E., Rosser T., Bilder R.M., Silva A.J., Bearden C.E. (2017). Spatial working memory in neurofibromatosis 1: Altered neural activity and functional connectivity. Neuroimage Clin. 15:801-811. doi: 10.1016/j.nicl.2017.06.032

Jernigan T.L., Bellugi U., Sowell E., Doherty S., Hesselink J.R. (1993). Cerebral morphologic distinctions between Williams and Down syndromes. Arch Neurol. 50(2):186-91. doi: 10.1001/archneur.1993.00540020062019.

Jung D.H., Ahn S.M., Pak M.E., Lee H.J., Jung Y.J., Kim K.B., Shin Y.I., Shin H.K., Choi B.T. (2020).Therapeutic effects of anodal transcranial direct current stimulation in a rat model of ADHD. Elife. 21;9:e56359. doi: 10.7554/eLife.56359

Kaufmann W.E. and Moser H.W. (2000). Dendritic anomalies in disorders associated with mental retardation. Cereb Cortex. 10(10):981-91. doi: 10.1093/cercor/10.10.981.

Kaufmann W.E., MacDonald S.M., Altamura C.R. (2016). Dendritic cytoskeletal protein expression in mental retardation: an immunohistochemical study of the neocortex in Rett syndrome. Cereb Cortex. 10(10):992-1004. doi: 10.1093/cercor/10.10.992.

Lazzaro G., Fucà E., Caciolo C., Battisti A., Costanzo F., Varuzza C., Vicari S., Menghini D. (2022). Understanding the Effects of Transcranial Electrical Stimulation in Numerical Cognition: A Systematic Review for Clinical Translation. J Clin Med. 11(8):2082. doi: 10.3390/jcm11082082.

Lazzaro G., Bertoni S., Menghini D., Costanzo F., Franceschini S., Varuzza C., Ronconi L., Battisti A., Gori S., Facoetti A., Vicari S. (2021). Beyond Reading Modulation: Temporo-Parietal tDCS Alters Visuo-Spatial Attention and Motion Perception in Dyslexia. Brain Sci. 19;11(2):263. doi: 10.3390/brainsci11020263

Leffa D.T., Bellaver B., Salvi A.A., de Oliveira C., Caumo W., Grevet E.H., Fregni F., Quincozes-Santos A., Rohde L.A., Torres I.L.S. (2018). Transcranial direct current stimulation improves long-term memory deficits in an animal model of attention-deficit/hyperactivity disorder and modulates oxidative and inflammatory parameters. Brain Stimul. 11(4):743-751. doi: 10.1016/j.brs.2018.04.001

Lehtonen A., Howie E., Trump D., Huson S.M. (2012). Behaviour in children with neurofibromatosis type 1: cognition, executive function, attention, emotion, and social competence. Dev Med Child Neurol. 55(2):111-125. doi: 10.1111/j.1469-8749.2012.04399.x

Molosh A.I., Johnson P.L., Spence J.P., Arendt D., Federici L.M., Bernabe C., Janasik S.P., Segu Z.M., Khanna R., Goswami C., Zhu W., Park S.J., Li L., Mechref Y.S., Clapp D.W., Shekhar A. (2014). Social learning and amygdala disruptions in Nf1 mice are rescued by blocking p21-activated kinase. Nat Neurosci. 17(11):1583-90. doi: 10.1038/nn.3822

Moyal M., Plaze M., Baruchet A., Attali D., Cravero C., Raffin M., Consoli A., Cohen D., Haroche A., Chaumette B. (2022). Efficacity of tDCS in catatonic patients with Phelan McDermid syndrome, a case series. Brain Stimul. 15(6):1432-1434. doi: 10.1016/j.brs.2022.10.005

Phelan K., Rogers R.C., Boccuto L. (1993). Phelan-McDermid Syndrome. In: GeneReviews®. University of Washington, Seattle, Seattle (WA).

Phelan K., McDermid H.E. (2011). The 22q13.3 Deletion Syndrome (Phelan-McDermid Syndrome). Mol Syndromol. 2(3-5):186-201. doi: 10.1159/000334260

Plasschaert E., Van Eylen L. (2016). Descheemaeker MJ, Noens I, Legius E, Steyaert J. Executive functioning deficits in children with neurofibromatosis type 1: The influence of intellectual and social functioning. Am J Med Genet B Neuropsychiatr Genet. 171B(3):348-62. doi: 10.1002/ajmg.b.32414.

Pobric G., Taylor J.R., Ramalingam H.M., Pye E., Robinson L., Vassallo G., Jung J., Bhandary M., Szumanska-Ryt K., Theodosiou L., Evans D.G., Eelloo J., Burkitt-Wright E., Hulleman J.,

Green J., Garg S. (2021). Cognitive and Electrophysiological Correlates of Working Memory Impairments in Neurofibromatosis Type 1. J Autism Dev Disord. 52(4):1478-1494. doi: 10.1007/s10803-021-05043-3

Poje A.B., Manzardo A., Gustafson K.M., Liao K., Martin L.E., Butler M.G. (2021). Effects of Transcranial Direct Current Stimulation (tDCS) on Go/NoGo Performance Using Food and Non-Food Stimuli in Patients with Prader-Willi Syndrome. Brain Sci. 11(2):250. doi: 10.3390/brainsci11020250

Rett A. (1966). U˙ber ein zerebral-atrophisches Syndrome bei Hyperam-monemie. Vienna: Bruder Hollinek

Ruohonen J., Karhu J. (2012). tDCS possibly stimulates glial cells. Clin Neurophysiol. 123(10):2006-9. doi: 10.1016/j.clinph.2012.02.082.

Salehinejad M.A., Ghanavati E., Glinski B., Hallajian A.H., Azarkolah A. (2022). A systematic review of randomized controlled trials on efficacy and safety of transcranial direct current stimulation in major neurodevelopmental disorders: ADHD, autism, and dyslexia. Brain Behav.12(9):e2724. doi: 10.1002/brb3.2724

Sarasua S.M., Boccuto L., Sharp J.L., Dwivedi A., Chen C.F., Rollins J.D., Rogers R.C., Phelan K., DuPont B.R. (2014). Clinical and genomic evaluation of 201 patients with Phelan-McDermid syndrome. Hum Genet. 133(7):847-59. doi: 10.1007/s00439-014-1423-7

Shilyansky C., Lee Y.S., Silva A.J. (2010). Molecular and cellular mechanisms of learning disabilities: a focus on NF1. Annu Rev Neurosci. 33:221-43. doi: 10.1146/annurev-neuro-060909-153215.

Schneider H.D. and Hopp J.P. (2011). The use of the Bilingual Aphasia Test for assessment and transcranial direct current stimulation to modulate language acquisition in minimally verbal children with autism. Clin Linguist Phon. 25(6-7):640-54. doi: 10.3109/02699206.2011.570852

Sousa B., Martins J., Castelo-Branco M., Gonçalves J. (2022). Transcranial Direct Current Stimulation as an Approach to Mitigate Neurodevelopmental Disorders Affecting Excitation/Inhibition Balance: Focus on Autism Spectrum Disorder, Schizophrenia, and Attention Deficit/Hyperactivity Disorder. J Clin Med. 18;11(10):2839. doi: 10.3390/jcm11102839

Vacca R.A., Bawari S., Valenti D., Tewari D., Nabavi S.F., Shirooie S., Sah A.N., Volpicella M., Braidy N., Nabavi S.M. (2019). Down syndrome: Neurobiological alterations and therapeutic targets. Neurosci Biobehav Rev. 98:234-255. doi: 10.1016/j.neubiorev.2019.01.001

Vacca R.A., Augello A., Gallo L., Caggianese G., Malizia V., La Grutta S., Murero M., Valenti D., Tullo A., Balech B., Marzano F., Ghezzo A., Tancredi G., Turchetta A., Riccio M.P., Bravaccio C., Scala I. (2023). Serious Games in the new era of digital-health interventions: A narrative review of their therapeutic applications to manage neurobehavior in neurodevelopmental disorders. Neurosci Biobehav Rev.;149:105156. doi: 10.1016/j.neubiorev.2023.105156

Van Steenburgh J.J., Varvaris M., Schretlen D.J., Vannorsdall T.D., Gordon B. (2017). Balanced bifrontal transcranial direct current stimulation enhances working memory in adults with high-

functioning autism: a sham-controlled crossover study. Mol Autism. 28;8:40. doi: 10.1186/s13229-017-0152-x

Vicari S., Bellucci S., Carlesimo G.A. (2005). Visual and spatial long-term memory: differential pattern of impairments in Williams and Down syndromes. Dev Med Child Neurol. (5):305-11. doi: 10.1017/s0012162205000599

Watt J.L., Olson I.A., Johnston A.W., Ross H.S., Couzin D.A., Stephen G.S. (1985). A familial pericentric inversion of chromosome 22 with a recombinant subject illustrating a 'pure' partial monosomy syndrome. J Med Genet. 22(4):283-7. doi: 10.1136/jmg.22.4.283

Zhang Y., Wang J., Zhang G., Zhu Q., Cai W., Tian J., Zhang Y.E., Miller J.L., Wen X., Ding M., Gold M.S., Liu Y. (2015). The neurobiological drive for overeating implicated in Prader-Willi syndrome. Brain Res. 1620:72-80. doi: 10.1016/j.brainres.2015.05.008
